# Target and Suspect HRMS Metabolomics for the Determination of Functional Ingredients in 13 Varieties of Olive Leaves and Drupes from Greece

**DOI:** 10.3390/molecules25214889

**Published:** 2020-10-22

**Authors:** Evangelia Kritikou, Natasa P. Kalogiouri, Lydia Kolyvira, Nikolaos S. Thomaidis

**Affiliations:** Laboratory of Analytical Chemistry, Department of Chemistry, National and Kapodistrian University of Athens, Panepistimiopolis Zographou, 15771 Athens, Greece; evkritik@chem.uoa.gr (E.K.); kalogiourin@chem.uoa.gr (N.P.K.); lydiakoli94@gmail.com (L.K.)

**Keywords:** metabolomics, olive leaves, drupes, suspect, LC-QTOF-MS, Greek, phenolics, biomarkers, oleuropein

## Abstract

The huge interest in the health-related properties of foods to improve health has brought about the development of sensitive analytical methods for the characterization of natural products with functional ingredients. Greek olive leaves and drupes constitute a valuable source of biophenols with functional properties. A novel ultra-high-performance liquid chromatography–quadrupole time of flight tandem mass spectrometry (UHPLC-QTOF-MS) analytical method was developed to identify biophenols through target and suspect screening in Greek olive leaves and drupes of the varieties: Koroneiki, Throumbolia, Konservolia, Koutsourelia, Kalamon, Petrolia, Amigdalolia, Megaritiki, Mastoeidis, Agouromanakolia, Agrilia, Adramitiani and Kolovi. The method’s performance was evaluated using the target compounds: oleuropein, tyrosol and hydroxytyrosol. The analytes demonstrated satisfactory recovery efficiency for both leaves (85.9–90.5%) and drupes (89.7–92.5%). Limits of detection (LODs) were relatively low over the range 0.038 (oleuropein)–0.046 (hydroxytyrosol) and 0.037 (oleuropein)–0.048 (hydroxytyrosol) for leaves and drupes, respectively For leaves, the precision limit ranged between 4.7% and 5.8% for intra-day and between 5.8% and 6.5% for inter-day experiments, and for drupes, it ranged between 3.8% and 5.2% for intra-day and between 5.1 and 6.2% for inter-day experiments, establishing the good precision of the method. The regression coefficient (r^2^) was above 0.99 in all cases. Furthermore, the preparation of herbal tea from olive leaves is suggested after investigating the optimum infusion time of dried leaves in boiling water. Overall, 10 target and 36 suspect compounds were determined in leaves, while seven targets and thirty-three suspects were identified in drupes, respectively.

## 1. Introduction

The olive tree (*Olea europaea* L.) is widely cultivated in many parts of the world [1]. Its cultivation dates back more than 7000 years. Nowadays, there are more than 2000 varieties in the Mediterranean basin [2], and specifically in Greece the number of cultivars is greater than 40 [3]. The climatic conditions in the Mediterranean countries, warm weather and sunlight irradiation favor the cultivation of the olive trees [1], while, at the same time, they activate the synthesis of phenolic compounds that are stored in dark fruits (olives) and thick leaves (olive leaves) [4].

Olive leaves are by-products of olive tree cultivation (pruning) and olive oil production [5,6,7,8]. The leaves of *O. europaea* L. are rich in phenols, characterized as “biophenols” since they present several therapeutic properties [6,7]. It is worth mentioning that the water extract of olive leaves has been widely used in traditional medicine to cure fever and treat several diseases such as hypertension, inflammation and diabetes as well [9]. Moreover, commercial products in the form of herbal teas or food supplements are available worldwide as dried leaves, powder, extracts or tablets [10]. It has been demonstrated that olive leaf extracts’ beneficial health properties are a consequence of the function of its biophenols [11]. More specifically, phenolic compounds have a broad spectrum of bioactive properties [2,12,13,14,15]. Based on previous reports, the most abundant functional compounds in olive leaves are oleuropein, tyrosol and hydroxytyrosol [16]. Even though oleuropein is mainly concentrated in olive leaves, it is also found in drupes [7]. Oleuropein is the major secoiridoid of the olive fruit [13], and its concentration decreases during fruit ripening, giving rise to hydroxytyrosol, which is the main product of oleuropein degradation, and to other simple phenols such as tyrosol [4,17,18]. Oleuropein has numerous beneficial health properties such as anti-inflammatory, cardioprotective, antioxidant, antiangiogenic, anti-cancer and neuroprotective functions, and thus may be of therapeutic potential for a variety of human disorders [11,19,20]. Tyrosol and hydroxytyrosol present significant antioxidant activity as well [21]. The huge interest in the health-related properties of foods and especially natural products has brought about the development of robust and sensitive analytical methodologies than can be applied for the determination of functional ingredients to enable the assessment of the molecular fingerprint of foods.

During the past decade, several works have been published for the determination of phenolic compounds in olive oils [22,23,24] and table olives [25], as has been recently reviewed [16]. However, the complete characterization of the phenolic profile of olive leaves still remains uninvestigated [13]. Only a few reports are available concerning the determination of phenolic compounds in Italian [7], Tunisian [8,26] and Ιranian *O. europaea* leaves [27]. As for the Greek olive leaves and drupes, limited data are available about minor constituents’ determination in Greek *O. europea* organs, olive oil, leaves and drupes, while there are no comparative studies investigating this topic [10,13,28,29]. Thus, there is an emerging need to develop analytical methods and analyze leaves and drupes to fill this gap in the literature.

The vast majority of the extraction protocols that have been reported for the determination of phenolic compounds in olive organs use mainly solid–liquid extraction (SLE) [1,7,8,21,26,27,30,31,32,33,34]. Solvents such as ethanol [14,19,35,36], water [14,19,28,36,37], acetone [28], ethyl acetate [13,36] and mixtures of ethanol:water [5,38,39] have been used for the extraction of biophenols in olives and leaves. However, the solvents mainly used are methanol [13,15,29,32,37] and mixtures of methanol:water (80:20, *v*/*v*) [1,8,31,33,34]. For a simple and rapid determination of total phenolic content in olive leaves and fruits, the most common method is the spectrophotometric Folin–Ciocalteu method [30,34,35]. Analytical techniques such as Nuclear Magnetic Resonance (NMR) [40] and gas chromatography (GC) coupled to mass spectrometry (MS) [32,33] or flame ionization detector (FID) [41] have also been reported. Although GC analysis results are very reliable, the step of derivatization makes this technique seldom applicable [21]. Due to good separation of metabolites, efficiency, versatility and short time of analysis, the most frequently used analytical technique for the identification and quantification of single phenolic compounds present in olive fruits and leaves is reversed-phase high/ultra-high performance liquid chromatography (RP-HPLC/UHPLC) [17,21] coupled to diode array detector (DAD) [13,28], MS [35,42] or MS/MS [19,43,44,45,46]. DAD is a timid alternative to MS, providing valuable and complementary ultraviolet–visible (UV–vis) spectra information but is far less sensitive than MS [47]. Therefore, LC-MS constitutes a potent analytical technique for the characterization of biophenols, particularly, using a mild ionization source like electrospray ionization (ESI) [19,44,48]. High-resolution mass spectrometry (HRMS) has been adopted to analyze complex matrices, benefiting from many aspects such as higher sensitivity, selectivity as well as higher mass accuracy [47]. HRMS analyzers mostly used for the determination of phenols in olive leaves and drupes are time of flight (TOF) [30,44,49], orbitrap [50] and hybrid mass analyzers such as quadrupole/TOF (QTOF) [19,46,47]. QTOF combines high sensitivity and mass accuracy for both precursor and product ions, providing the parent and fragment ions’ elemental composition [19]. LC-HRMS enables the identification of a wide range of analytes through target and non-target screening strategies, as has already been reviewed [16], and can be successfully applied in the phenolic fingerprinting of *O. europaea* L. leaves and drupes.

The primary purpose of this study was to develop and validate target and suspect UHPLC-QTOF-MS/MS methodologies for the determination of phenolic compounds in Greek olive leaves and drupes that belonged to the varieties: Koroneiki, Throumbolia, Konservolia, Koutsourelia, Kalamon, Petrolia, Amigdalolia, Megaritiki, Mastoeidis, Agouromanakolia, Agrilia, Adramitiani and Kolovi. For this purpose, 47 samples of olive leaves and 15 samples of drupes were acquired from different regions in Greece. A target screening protocol was followed for the determination of the target compounds, and, in a further step, oleuropein, hydroxytyrosol and tyrosol were quantified in both matrices. The samples’ quantification results were compared by creating box and whisker plots and performing one-way analysis of variance (ANOVA). A suspect screening workflow was applied for the identification of 60 bioactive compounds from an initial suspect list that was built from the literature. Characteristic spectra of the identified suspect compounds are presented, and their fragments are explained. Finally, the tea preparation protocol of infused olive leaves in boiling water was optimized after monitoring the concentration levels of oleuropein, tyrosol and hydroxytyrosol in different periods of infusion (3, 6 and 10 min).

## 2. Materials and Methods

### 2.1. Chemicals and Reagents

Methanol (LC-MS grade) and sodium hydroxide (>99%) were purchased from Merck (Darmstadt, Germany). Ammonium acetate (≥99% for HPLC) and formic acid (LC-MS Ultra) were purchased from Fluka (Buchs, Switzerland). Isopropanol was obtained from Fisher Scientific (Geel, Belgium). Ultrapure water was provided by a Milli-Q purification apparatus (Millipore Direct-Q UV, Bedford, MA, USA). Regarding the standards that were used, syringic acid 95% was purchased from Extrasynthèse (Genay, France). Gallic acid 98%, ferulic acid 98%, epicatechin 97%, *p*-coumaric (4-hydroxycinnamic acid) 98%, homovanillic acid 97%, quercetin 98%, oleuropein 98%, pinoresinol 95%, caffeic acid 99%, taxifolin 98%, vanillic acid 97% and syringaldehyde 98% (internal standard) were obtained from Sigma-Aldrich (Steinheim, Germany). Hydroxytyrosol 98% and luteolin 98% were acquired from Santa Cruz Biotechnologies. Vanillin 99%, apigenin (4,5,7-trihydroxyflavone) 97% and tyrosol (2-(4-hydroxyphenyl) ethanol) 98% were purchased from Alfa Aesar (Karlsruhe, Germany). Stock standard solutions of individual compounds (1000 mg/L) were solubilized in methanol and stored at −20 °C in dark brown glass bottles. Intermediate standard working solutions containing the analytes were prepared by appropriate dilution of the stock solutions with methanol:water (80:20, *v*/*v*) over the concentration range 0.1–8 mg/L.

### 2.2. Instrumentation

A UHPLC system with a HPG-3400 pump (Dionex UltiMate 3000 RSLC, Thermo Fisher Scientific, Germany) coupled to a QTOF mass spectrometer (Maxis Impact, Bruker Daltonics, Bremen, Germany) was used. The separation was performed using an Acclaim RSLC C18 column (2.1 × 100 mm, 2.2 μm) purchased from Thermo Fisher Scientific (Driesch, Germany) with an ACQUITY UPLC BEH C18 precolumn (1.7 μm, VanGuard precolumn, Waters, Ireland). Column temperature was set at 30 °C. The solvents used consisted of (A) 90% water, 10% methanol and 5 mM CH_3_COONH_4_, (B) 100% methanol and 5 mM CH_3_COONH_4_. The elution program was gradient and started with 1% of B with a flow rate of 0.2 mL/min for 1 min, gradually increasing to 39% in the next 2 min, and then increasing to 99.9% and a flow rate of 0.4 mL/min for the following 11 min. These conditions were kept constant for 2 min (flow rate 0.48 mL/min,) and then the initial conditions (99%A, 1%B) were restored within 0.1 min (flow rate decreased to 0.2 mL/min) for re-equilibration of the column.

The QTOF MS system was equipped with an ESI interface, operating in a negative mode with the following settings: capillary voltage of 3500 V, end plate offset of 500 V, nebulizer pressure of 2 bar (N_2_), drying gas flow rate of 8 L/min (N_2_) and drying temperature of 200 °C. External calibration was performed daily with a sodium formate cluster solution consisting of 10 mM sodium formate in a mixture of water/isopropanol (1:1, *v*/*v*). Additionally, the calibration solution was injected at the beginning of each run, and a segment (0.1−0.25 min) in every chromatogram was used for internal calibration. Full scan mass spectra were recorded in the range from 50 to 1000 *m*/*z*, with a scan rate of 2 Hz. MS/MS experiments were conducted using data-dependent acquisition (AutoMS, otofControl, Bruker Daltonics, Bremen, Germany) mode based on the fragmentation of the five most abundant precursor ions per scan. For certain compounds of interest whose intensity of the *m*/*z* was low, a second analysis including a list of the selected precursor ions was performed in AutoMS mode. The instrument provided a typical resolving power (full width at half maximum) between 36,000 and 40,000 at *m*/*z* 226.1593, 430.9137, and 702.8636.

### 2.3. Sampling and Sample Preparation

**Figure 1:**
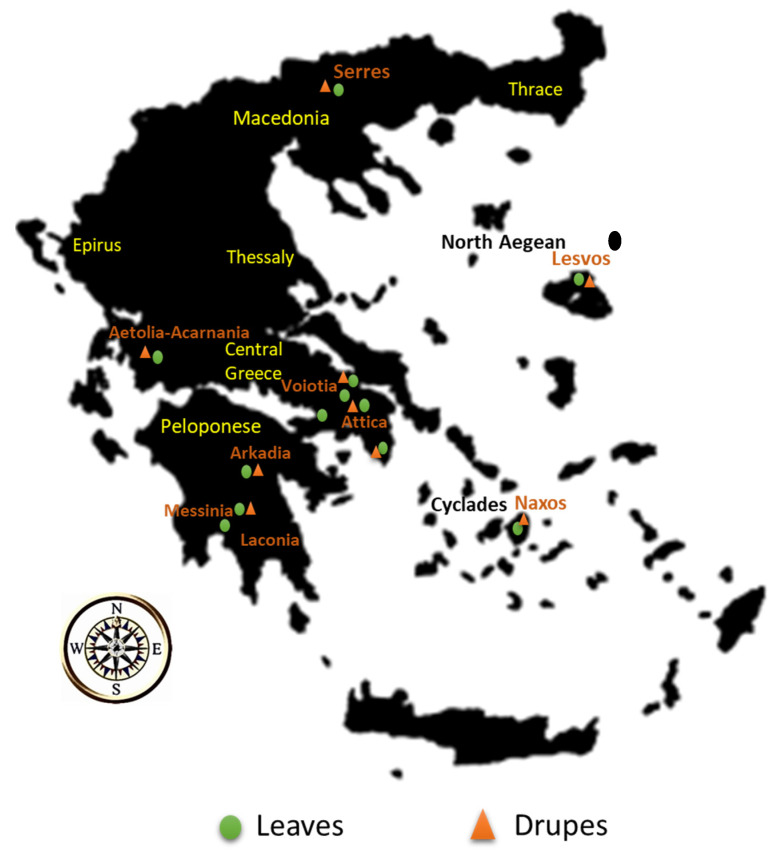
Geographical origin of olive leaves and drupes.

Forty-seven leaves and 15 drupe samples belonging to the varieties Koroneiki, Throumbolia, Konservolia, Koutsourelia, Kalamon, Petrolia, Amigdalolia, Megaritiki, Mastoeidis, Agouromanakolia, Agrilia, Adramitiani and Kolovi were acquired from local producers from various regions in Greece during the harvesting period December 2016–January 2017. All the samples acquired were cultivated with organic type of farming. Figure 1 illustrates the geographical origin of the leaves and drupes acquired, and Table S1 in the Electronic Supplementary Material presents the number of the samples acquired along with the data for each sample (variety, geographical origin).

All the collected samples (leaves and drupes) were processed within the same day that they were received. First, all samples were washed with water; then, the branches were removed from the leaves, and the cores were removed from the drupes accordingly. SLE was used in order to isolate the phenolic compounds from leaves and drupes. Each sample was blended using a laboratory blade cutter. Then, 0.5 g of homogenous sample was weighed in a 2-mL Eppendorf tube, spiked with 1 mg/L syringaldehyde, and, afterwards, 1 mL of extraction solvent methanol:water (80:20, *v*/*v*) was added. In the following step, the mixture was vortexed for 2 min and centrifuged for 5 min at 13,400 rpm. The upper phase (extract) was collected and filtered through membrane syringe filters of regenerated cellulose (CHROMAFIL^®^ RC) (15 mm diameter, 0.22 μm pore size, purchased from MachereyNagel, Düren, Germany). The extracts were stored at −20 °C prior to analysis. Finally, 5 μL of this solution was injected into the chromatographic system.

### 2.4. Quality Control

Quality control (QC) samples were used to verify that the analytical equipment remained stable during the analysis of samples’ batch. The QC samples were prepared by mixing all aliquots of all the samples under study. At the beginning of the analysis, the QC sample was injected five times for conditioning, and afterwards it was injected at regular intervals (every ten sample injections) throughout the analytical run in order to ensure the good performance of analytical equipment and, consequently, the validity of the results. The relative standard deviations (RSD) for the retention time (t_R_), peak areas and Δm errors (*n* = 11) of the target compounds (oleuropein, tyrosol, hydroxytyrosol) are presented in the Electronic Supplementary Material (Table S2), proving the good performance of the analytical system. Procedural blanks were also prepared and processed in the chromatographic system to detect any potential contamination during the analysis.

### 2.5. Target Screening

Among olive metabolites, phenolic compounds constitute a broad class of biomarkers. Considering that the qualitative and quantitative phenolic composition of olive fruits and leaves depend on the cultivar [19,36,51], the geographical location [17,21,52], and the climatic and environmental conditions [21,36,53], our study was focused on the detection and identification of phenolic compounds of different classes.

For this, a target list of 15 phenols with commercially available standards was built. These 15 compounds have already been identified in olive leaves and drupes, as well as in other organs of *O. europaea* L. (bark, seeds, stem and root) and olive oil according to the literature [2,13,19,21,44,47,50,54]. The initial target list (Table S3) constituted of caffeic acid, ferulic acid, gallic acid, homovanillic acid, *p*-coumaric acid and syringic acid from the class of phenolic acids; hydroxytyrosol and tyrosol from the class of phenolic alcohols; vanillin which is a phenolic aldehyde; apigenin, epicatechin, quercetin and luteolin from the class of flavonoids; the secoiridoid oleuropein; and pinoresinol from the class of lignans.

Target screening was performed using Bruker softwares (Bruker Daltonics, Bremen, Germany) TargetAnalysis 1.3 and DataAnalysis 4.1, as well as tools available in these software (Bruker Compass IsotopePattern and Smart Formula Manually). Extracted Ion Chromatograms (EICs) were obtained, and the following parameters were implemented: mass accuracy threshold up to 5 ppm, isotopic fit below or equal to 100 mSigma (mSigma is a measure for the goodness of fit between measured and theoretical isotopic pattern), signal-to-noise (S/N) threshold was set at 3, minimum peak area threshold was set at 2000, and minimum ion intensity threshold was set at 500. Relative tolerance of the retention time window was set lower than ±0.2 min. All target compounds were identified based on mass accuracy, isotopic pattern, retention time (t_R_) and MS/MS fragments.

### 2.6. Method Validation

The developed RP-UHLPC-QTOF-MS/MS methodology was validated to verify its suitability for identification and quantification purposes. Considering that oleuropein, tyrosol and hydroxytyrosol are the most abundant biophenols in olive tree organs, the validation was performed using homogenized leaf and drupe samples which were spiked with a mix of these three biophenols over the range 0.1–8 mg/Kg (10 calibration levels with 3 replicates at each level). The calibration curves were constructed using the peak area of the spiked target compound subtracted by the peak area of the neat sample and divided by the peak area of the internal standard. The limits of detection (LODs) and method limits of quantification (LOQs) were defined as the analyte’s concentration at which the signal-to-noise ratio (S/N) was above 3 and 10, respectively. The accuracy of the method was estimated using recoveries at a 1 mg/Kg concentration level calculated with the following equation:

(1) $$\%\mathrm{RE}=\;\frac{Response\;extracted\;sample}{Response\;of\;the\;post-extracted\;spike\;sample}$$

where response extracted sample is the average area from 3 replicates of the analyte in matrix, which has been through the extraction process divided by the area of the internal standard (syringaldehyde 1 mg/mL). The response of the post-extracted spike sample is the average area of each analyte spiked into the already extracted matrix.

The matrix effect was calculated at the 1 mg/Kg concentration level by comparing analytes’ standard solutions in pure solvent and in matrix-matched samples according to the following equation:

(2) $$\%\mathrm{ME}=\;\left(\frac{Response\;matrix\;matched\;sample}{Response\;of\;the\;standard\;in\;pure\;solvent}-1\right)\times 100$$

The precision was expressed in terms of repeatability (intra-day precision) and intermediate precision (inter-day precision) at spiked concentrations of 1 mg/Kg of syringaldehyde. Repeatability was expressed as the %RSD_r_ values of 10 replicate analyses (*n* = 10) in the same day. Reproducibility experiments expressed as the %RSD_R_ value of 3 replicates of two consecutive days (n × k = 3 × 2 = 6).

### 2.7. Suspect Screening

A suspect list was generated from the literature consisting of 60 significant bioactive compounds that have been identified in olive leaves, drupes, other organs of *O. europaea* L. and olive oil [2,13,17,19,21,37,44,47,48,50,55,56] in order to investigate their presence in the samples. The initial suspect list is presented in Table S4.

For the identification of the suspect compounds, the masses of the deprotonated ions were calculated based on their molecular formula using the Isotope Pattern tool (Bruker software, Bruker Daltonics, Bremen, Germany), and EICs were created in DataAnalysis 4.1 (Bruker software, Bruker Daltonics, Bremen, Germany). The parameters implemented in this case were the following: mass accuracy threshold up to 5 ppm, isotopic fit below or equal to 100 mSigma, minimum peak area threshold was set to 2000, minimum ion intensity threshold was set to 800, and peak score (ratio peak area/intensity) was more than 4 (preferable peak score should be between 4–38 [57]). The MS/MS fragments were compared and verified using in silico fragmentation tools, such as MetFrag [58] and data reported in previous studies. The experimental t_R_ of each compound was compared with the predicted t_R_ by an in-house model based on the Quantitative Structure–Retention Relationship (QSRR) [59]. The experimental t_R_ of the compounds that belong to the suspect list have already been predicted and included in a database consisting of 1606 commonly occurring compounds in various olive matrices, which our group has already published [23]. For communication reasons, the level of confidence achieved during the identification of the suspect compounds was established according to Schymanski et al. [60]. Identification level 1 corresponds to the confirmation of the suspect compounds with a reference standard. Level 2 includes two sublevels: level 2a if there is evidence matching MS/MS information with spectral libraries or literature data and level 2b if there is diagnostic evidence, such as the agreement between experimental and predicted t_R_. Level 3 corresponds to tentative candidates.

### 2.8. Statistical Analysis

Statistical analysis was performed using analysis of variance (ANOVA) from Data Analysis tool of Microsoft Excel. Generally, ANOVA is utilized to examine if there are significant statistical differences between the variances of independent groups of data. In the present work, ANOVA was used for the comparison of the results between different varieties. Results were tested for statistically significant differences by one-way ANOVA. For the evaluation of the results, *p*-value was used for confidence level 95%. If the *p*-value is higher than 0.05, there is no significant statistical difference. On the contrary, if the *p*-value is lower than 0.05, there is significant statistical differences between the studied samples.

### 2.9. Olive Leaf Tea Preparation

Leaves were collected and immediately washed and dried physically in air atmosphere and sunlight. The dry leaves were ground using a laboratory mixer. A total of 1g of the powdered sample was added in an empty tea bag and infused in 100 mL of boiling water for 3, 6 and 10 min to monitor the alterations in the concentrations of oleuropein, tyrosol and hydroxytyrosol during the different boiling times. The infusions were left at room temperature to cool and were filtered through regenerated cellulose 0.22 μm filters. An amount of 1 mL of each infusion sample was spiked with syringaldehyde at a final concentration of 1 mg/L and was processed in the chromatographic system. The quantification of the target analytes was carried out using normalized standard calibration curves over the concentration range of 1–8 mg/Kg (equations: y = (a ± Sa) + (b ± Sb)x; oleuropein: y = (3.04 ± 0.39) + (1.73 ± 0.08)x; tyrosol: (1.30 ± 0.09) + (0.35 ± 0.02)x; hydroxytyrosol: y = (2.80 ± 0.20) + (0.97 ± 0.04)x.

## 3. Results and Discussion

### 3.1. Method Development and Validation Results for Leaves

The analytes demonstrated satisfying recovery efficiency (85.9–90.5%). The precision limit ranged between 4.7–5.8% for intra-day experiments and between 5.8–6.5% for inter-day experiments, establishing the good precision of the analytical method. The regression coefficient (r^2^) was above 0.99 in all cases. The validation parameters, including LODs and LOQs, RE, regression equations, regression coefficient (r^2^), intra-day and inter-day precision and the matrix effect are summarized in Table 1.

### 3.2. Method Development and Validation Results for Drupes

The validation parameters for the RP-UHPLC-QTOF-MS/MS method for drupes are summarized in Table 2. Oleuropein, tyrosol and hydroxytyrosol demonstrated satisfying recovery efficiency (89.7–92.5%). The precision limit ranged between 3.8–5.2% for intra-day experiments and between 5.1–6.2% for inter-day experiments, establishing the good precision of the analytical method. The regression coefficient (r^2^) was above 0.99 in all cases.

### 3.3. Determination of Bioactive Compounds in Leaves

#### 3.3.1. Target Screening Results for Leaves

From the initial target list of 15 compounds, 10 biophenols were determined in leaves. Those were ferulic acid and gallic acid from the group of phenolic acids, hydroxytyrosol and tyrosol from phenolic alcohols, the phenolic aldehyde vanillin, apigenin, luteolin and quercetin from the class of flavonoids, the secoiridoid oleuropein and the lignan pinoresinol. Oleuropein and luteolin were detected in all leaf samples. Ferulic acid was detected in the varieties Koroneiki from Naxos-Melanes, Konservolia from Naxos-Melanes, Koutsourelia from Aetolia-Acarnania-Agrinio, Kalamon from Aetolia-Acarnania-Agrinio, Petrolia from Serres-Skoutari, Amigdalolia from Attica-Votanikos, Kalamon from Attica-Votanikos, Konservolia from Attica-Votanikos, Koroneiki from Attica-Votanikos, Koroneiki from Messenia-Kalamata, Mastoeidis from Laconia-Sparti, Agouromanakolia form Laconia-Sparti, Agrilia from Laconia-Sparti, Koroneiki from Arcadia-Kynouria, Kolovi from Lesvos-Palaiohori, Moria, Adramitiani from Lesvos-Kalloni and Agrilia from Lesvos-Komi. Gallic acid was detected in the varieties Konservolia from Aetolia-Acarnania-Agrinio, Megaritiki from Attica-Megara and Megaritiki from Boeotia-Dilesi. Hydroxytyrosol was detected in all leaf varieties except for Agouromanakolia from Arcadia-Kynouria. Tyrosol was detected in all leaf varieties except for Petrolia from Serres-Skoutari, Mastoeidis from Laconia-Sparti, and Agouromanakolia from Arcadia-Kynouria. Vanillin was detected in all leaf varieties except for Agouromanakolia and Koroneiki from Arcadia-Kynouria. Apigenin was detected in all leaf varieties except for Koroneiki from Messenia-Kalamata, Agouromanakolia from Arcadia-Kynouria, Koroneiki from Arcadia-Kynouria and Koroneiki from Boeotia-Dilesi. Quercetin was detected in the varieties Throumbolia from Naxos-Melanes, Konservolia from Aetolia-Acarnania-Agrinio, Kalamon from Aetolia-Acarnania-Agrinio, Petrolia from Serres-Skoutari, Amigdalolia from Attica-Votanikos, Kalamon from Attica-Votanikos, Konservolia from Attica-Votanikos, Kalamon from Messenia-Kalamata, Megaritiki from Attica-Sounio, Megaritiki from Attica-Megara and Agouromanakolia form Laconia-Sparti. Pinoresinol was detected in the varieties Konservolia from Aetolia-Acarnania-Agrinio and Petrolia from Serres-Skoutari. The target screening results in leaves are presented in Table 3.

#### 3.3.2. Quantification Results for Leaves

**Figure 2:**
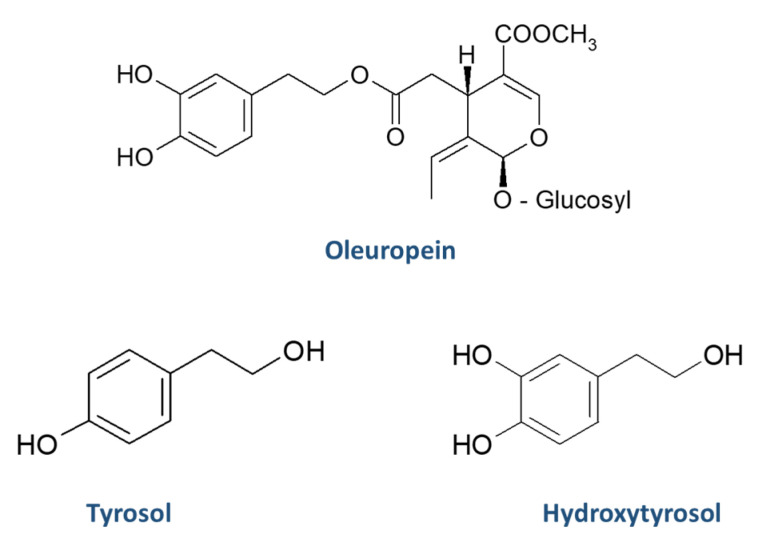
Chemical structures of oleuropein, tyrosol, and hydroxytyrosol.

The quantification of oleuropein, tyrosol and hydroxytyrosol was carried out using the calibration curves that were constructed, as described in Section 2.6. The quantification results in leaf samples of oleuropein, tyrosol and hydroxytyrosol (expressed in mg/Kg) are presented in Table 4. The chemical structures of oleuropein, tyrosol and hydroxytyrosol are illustrated in Figure 2. The concentration of oleuropein in the leaves ranged between below the LOQ (Agouromanakolia from Arcadia-Kynouria) and 293 mg/Kg (Agrilia from Laconia-Sparti). Tyrosol was in the range below the LOD (Petrolia from Serres-Skoutari, Mastoeidis from Laconia-Sparti and Agouromanakolia from Arcadia-Kynouria) and 121 mg/Kg (Kolovi from Lesvos-Palaiohori). The concentration of hydroxytyrosol was in the range below the LOD (Agouromanakolia from Arcadia-Kynouria) and 421 mg/Kg (Agrilia from Lesvos-Komi). Based on the sum of the concentrations of oleuropein, tyrosol and hydroxytyrosol, Agrilia from Lesvos-Komi had the highest phenolic content among the samples, while Kolovi from Lesvos-Palaiohori is ranked second.

Descriptive statistic (box and whisker plot) and statistical analysis (ANOVA) were performed for the varieties whose number of samples was equal or higher than three (*n* ≥ 3). For leaves, the varieties Koroneiki, Konservolia, Kalamon, Petrolia, Megaritiki, Agouromanakolia, Agrilia, Adramitiani and Kolovi were compared, and the concentrations of oleuropein, tyrosol and hydroxytyrosol were examined.

##### Oleuropein

**Figure 3:**
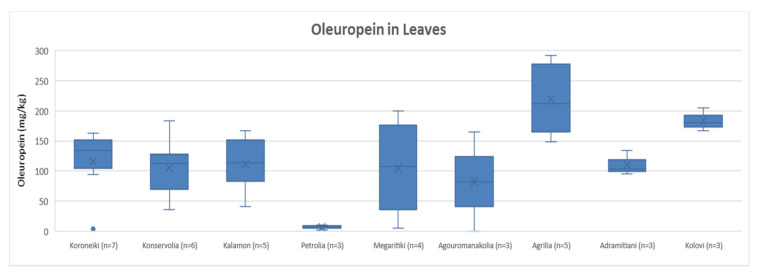
Box and whisker plot for the concentrations of oleuropein among different varieties of leaves.

After comparing the variances of oleuropein content in leaves, it was concluded that the varieties Koroneiki, Konservolia, Kalamon, Megaritiki, Agouromanakolia, Adramitiani and Kolovi had no statistically significant difference. The ANOVA showed a *p*-value > 0.05 (*p* = 0.53). On the other hand, Petrolia presented a statistically significant difference compared to Koroneiki, Konservolia, Kalamon, Agrilia, Adramitiani and Kolovi, since ANOVA demonstrated a *p*-value < 0.05. Moreover, as shown in Figure 3, Petrolia presented the lowest concentration of oleuropein on average (6.4 mg/Kg). Agrilia had the highest concentration of oleuropein on average (219 mg/Kg) and showed significant statistical difference among the other varieties (*p* < 0.05), except for the varieties Kolovi and Megaritiki. Furthermore, according to Figure 3, Kolovi presented the second highest oleuropein’s concentration on average (184 mg/Kg), while oleuropein concentration ranged from 82 mg/Kg to 116 mg/Kg among the other varieties.

##### Tyrosol

**Figure 4:**
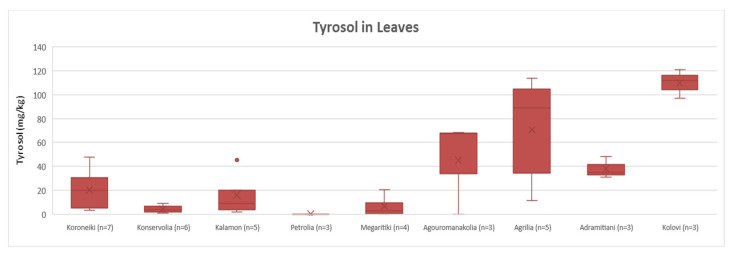
Box and whisker plot for the concentrations of tyrosol among different varieties of leaves.

Regarding the quantification results of tyrosol in leaves, Figure 4 shows that the varieties could be divided into two groups: one group with Agouromanakolia, Agrilia, Adramitiani and Kolovi with concentrations of tyrosol over the range 38–110 mg/Kg; and another group with Koroneiki, Konservolia, Kalamon and Megaritiki that presented lower concentrations (<LOQ–20 mg/Kg). This discrimination was in agreement with the results of the statistical analysis. ANOVA showed that Koroneiki, Konservolia, Kalamon and Megaritiki had no statistically significant difference (*p* = 0.17), while Agouromanakolia, Agrilia, Adramitiani and Kolovi did not differ statistically (*p* = 0.10) either. Moreover, the comparison of the two groups showed a significant statistical difference between them (*p* < 0.05). Petrolia was not included in the comparison since the concentration of tyrosol was below the LOD. Based on Figure 4, it is observed that Kolovi presented very high concentrations of tyrosol in all samples (97–121 mg/Kg).

##### Hydroxytyrosol

**Figure 5:**
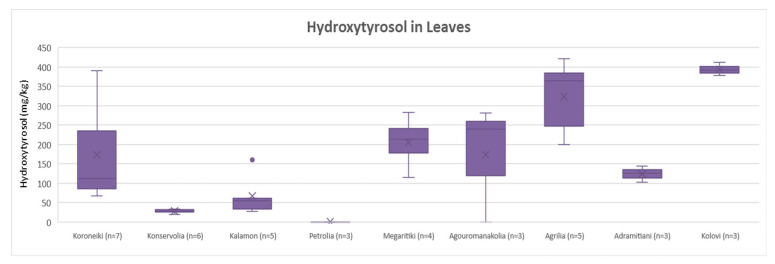
Box and whisker plot for the concentrations of hydroxytyrosol among different varieties of leaves.

Concerning the concentration of hydroxytyrosol in leaves, ANOVA showed no significant statistical difference for Konservolia and Kalamon (*p* = 0.10). Moreover, the varieties Koroneiki, Megaritiki, Agouromanakolia and Adramitiani did not differ statistically (*p* = 0.82). Comparing Agrilia with the other varieties, ANOVA presented significant statistical differences only with Adramitiani (*p* < 0.05). Kolovi showed a statistically significant difference compared with the other varieties, with the exception of Agrilia (*p* = 0.26). Petrolia was not included in the statistical analysis because the concentrations of tyrosol in the samples were below the LOQ. Figure 5 shows that the Kolovi variety presented the highest hydroxytyrosol concentration on average (394 mg/Kg), followed by Agrilia (323 mg/Kg). On the other hand, the varieties Konservolia and Kalamon presented lower concentrations of hydroxytyrosol (28 mg/Kg and 67 mg/Kg, respectively). The average concentrations of hydroxytyrosol in Koroneiki, Megaritiki, Agouromanakolia and Adramitiani ranged between 124 and 206 mg/Kg.

#### 3.3.3. Suspect Results for Leaves

In leaves, 36 bioactive compounds were identified from the initial suspect list of 60 compounds. The presence of vanillic acid and taxifolin was verified with the use of commercial standards. 4-hydroxybenzoic acid, 4-hydroxyphenylacetic acid, verbascoside, calceolarioside, hydroxytyrosol acetate, hydroxytyrosol glucoside, tyrosol glucoside (or salidroside), apigenin-7-*O*-glucoside, chrysoeriol (or luteolin 3′-methyl ether), diosmin, fustin, luteolin-7-*O*-glucoside, luteolin-7,4′-*O*-diglucoside, naringenin, quercetin-3-*O*-rutinoside (or rutin), vicenin-2, decarboxymethyl oleuropein aglycone (or oleacein), fraxamoside, ligstroside, ligstroside aglycone, oleoside, oleuropein aglycone, secologanoside, maslinic acid, oleanolic acid, aesculin, elenolic acid, elenolic acid 2-*O*-glucoside, and licodione were identified at level 2a. Demethyl oleuropein, 10-hydroxy-10-methyl oleuropein aglycone, 10-hydroxy decarboxymethyl oleuropein aglycone and 10-hydroxy oleuropein aglycone were identified at level 2b. Finally, the hydroxylated form of elenolic acid was identified at level 3. The suspect screening results are summarized in Table S5, providing information about the identification criteria and the level of identification of each compound.

**Figure 6:**
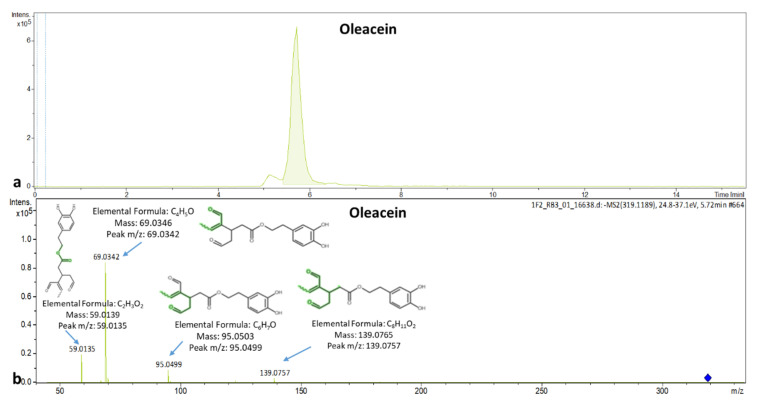
(**a**) Extracted ion chromatogram of oleacein in a leaf sample; (**b**) MS/MS spectrum of oleacein with explained fragments.

The cardiovascular effects of olive leaves are attributed to oleuropein and oleacein. The EIC chromatogram of oleacein and the spectrum with its explained fragments are presented in Figure 6a,b, respectively.

**Figure 7:**
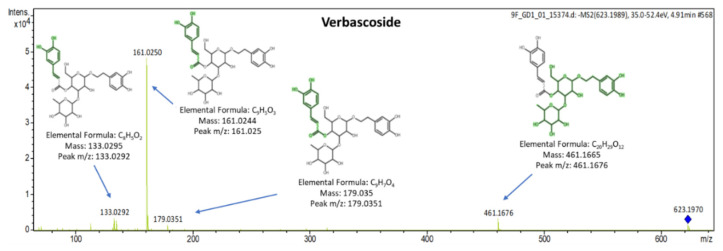
MS/MS spectrum of verbascoside identified in leaves with explained fragments.

Verbascoside is reported two or three times more active radical scavenger compared to hydroxytyrosol. The qualifier ions of verbascoside (t_R_ = 5 min) were detected at *m*/*z*: 113.0242, 133.0292, 153.0566, 161.0250, 179.0351, 241.0714, 275.0569, 315.1109 and 461.1676 corresponding to C_5_H_5_O_3_, C_8_H_5_O_2_, C_8_H_9_O_3_, C_9_H_5_O_3_, C_9_H_7_O_4_, C_11_H_13_O_6_, C_14_H_11_O_6_, C_14_H_19_O_8_, C_20_H_29_O_12._ The MS/MS spectrum of verbascoside is presented in Figure 7.

**Figure 8:**
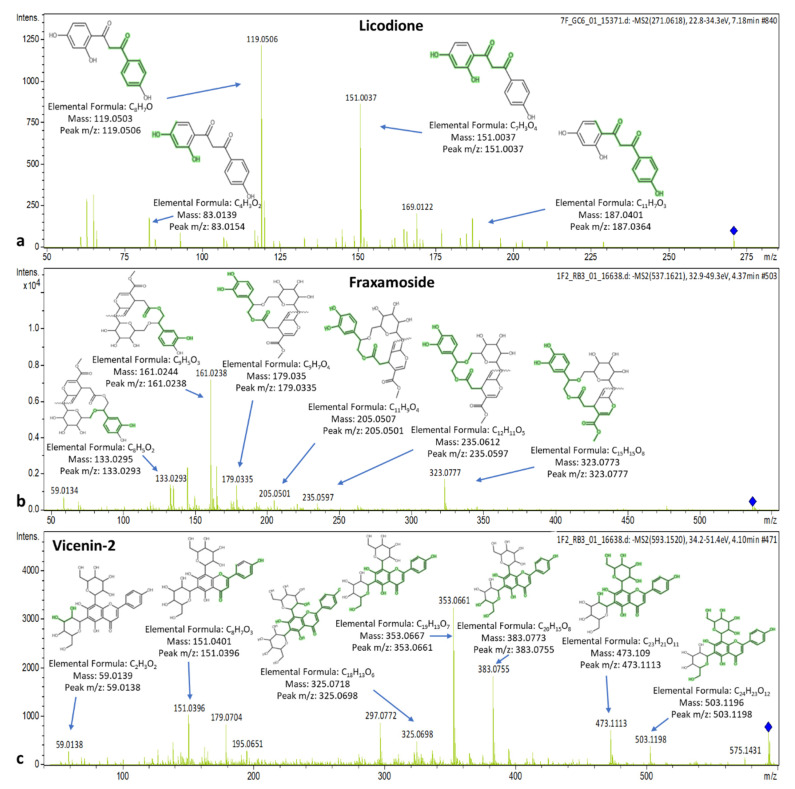
Spectra and MS/MS fragments of (**a**) licodione, (**b**) fraxamoside, and (**c**) vicenin-2.

The MS/MS spectrum of licodione presents peaks (t_R_ = 7.19 min) at *m*/*z*: 83.0154, 119.0506, 151.0037, 177.0160, and 187.0364 that correspond to C_4_H_3_O_2_, C_8_H_7_O, C_7_H_3_O_4_, C_9_H_5_O_4_ and C_11_H_7_O_3_, respectively. Fraxamoside elutes at 4.35 min presenting qualifier ions with *m*/*z*: 133.0293, 161.0238, 165.0547, 179.0335, 205.0501, 221.0472, 235.0597 and 323.0777 corresponding to C_8_H_5_O_2,_ C_9_H_5_O_3,_ C_9_H_9_O_3,_ C_9_H_7_O_4,_ C_11_H_9_O_4_, C_11_H_9_O_5_, C_12_H_11_O_5_ and C_15_H_15_O_8,_ respectively. Vicenin-2 elutes at 4.12 min and shows peaks at *m*/*z*: 151.0396, 325.0698, 353.0661, 383.0755, 413.0889, 473.1113, 503.1198 and 575.1431 that correspond to C_8_H_7_O_3_, C_18_H_13_O_6_, C_19_H_13_O_7_, C_20_H_15_O_8_, C_21_H_17_O_9_, C_23_H_21_O_11_, C_24_H_23_O_12_ and C_27_H_27_O_14_, respectively. The spectra with the MS/MS-explained fragments of licodione, fraxamoside, and vicenin-2 are presented in Figure 8.

**Figure 9:**
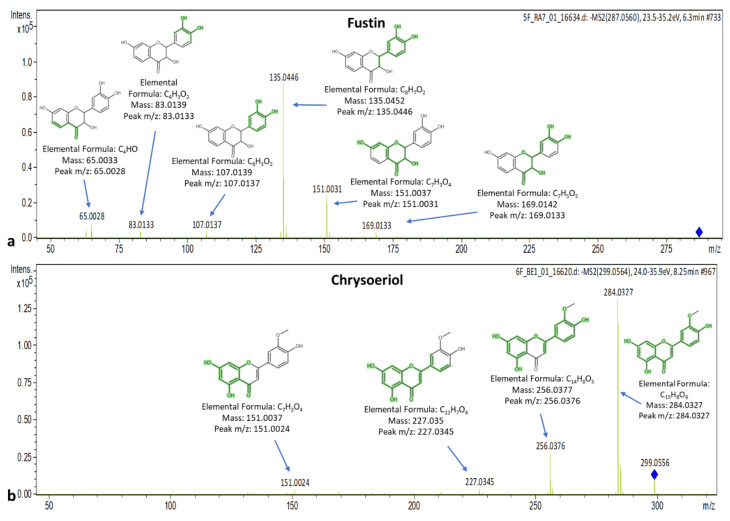
Spectra and MS/MS fragments of (**a**) fustin and (**b**) chrysoeriol.

Fustin elutes at 6.27 min, and its spectrum presents qualifiers ions with *m*/*z*: 83.0133, 107.0137, 125.0240, 135.0446, 151.0031, 161.0236, 169.0133, 177.0181, 185.0252, 203.0352 and 269.0462 that correspond to C_4_H_3_O_2_, C_6_H_3_O_2_, C_6_H_5_O_3_, C_8_H_7_O_2_, C_7_H_3_O_4_, C_9_H_5_O_3_, C_7_H_5_O_5_, C_9_H_5_O_4_, C_11_H_5_O_3_, C_11_H_7_O_4_ and C_15_H_9_O_5_, respectively. Chrysoeriol (t_R_ = 8.24 min) presents peaks at *m*/*z*: 83.0138, 107.0139, 134.0352, 151.0024, 158.0369, 200.0464, 227.0345, 256.0376 and 284.0327 that correspond to C_4_H_3_O_2_, C_6_H_3_O_2_, C_8_H_6_O_2_, C_7_H_3_O_4_, C_10_H_6_O_2_, C_12_H_8_O_3_, C_13_H_7_O_4_, C_14_H_8_O_5_ and C_15_H_8_O_6_, respectively. The spectra of fustin and chrysoeriol are presented in Figure 9.

Most of the compounds were detected in the varieties Koroneiki and Throumbolia from Naxos-Melanes, Koutsourelia from Aetolia-Acarnania-Agrinio, Kalamon from Aetolia-Acarnania-Agrinio and Attica-Votanikos, Amigdalolia and Konservolia from Attica-Votanikos. The Agouromanakolia and Koroneiki variety from Arcadia-Kynouria were found to have the fewest bioactive compounds compared to the other samples. The compounds detected in the majority of leaves (*n* ≥ 35) were 10-hydroxy-10-methyl oleuropein aglycone, elenolic acid and elenolic acid-2-*O*-glucoside, 10-hydroxy oleuropein aglycone, 4-hydroxyphenylacetic acid, hydroxytyrosol acetate, oleoside, oleuropein aglycone and secologanoside. On the other hand, the compounds detected in the minority of leaves (*n* ≤ 18) were diosmin, fustin and the hydroxylated form of elenolic acid. The latter was detected only in the varieties Koroneiki and Throumbolia from Naxos-Melanes, Koutsourelia from Aetolia-Acarnania-Agrinio and Mastoeidis from Laconia-Sparti.

#### 3.3.4. Determination of Phenolic Compounds in Tea Extracts Prepared under Different Temperatures

Agrilia leaves from Lesvos with the richest phenolic fingerprint in oleuropein, tyrosol and hydroxytyrosol were dried and infused in boiling water for 3, 6 and 10 min, in triplicate (*n* = 3). According to the results, it was observed that the increase in infusion of the olive leaves in boiling water from 3 to 10 min resulted in increased analytical signal of all the target and suspect bioactive compounds. Therefore, it could be concluded that by increasing the boiling time, the phenolic and other bioactive components of leaf are extracted in boiling water to a greater extent. Exceptions were the compounds luteolin, diosmin and calceolarioside, as their signal decreased by extending the boiling time. Moreover, some compounds had no significant differences with increasing boiling time. Those were gallic acid, vanillic acid, 4-hydroxybenzoic acid, fraxamoside, 10-hydroxy-10-methyl oleuropein aglycone and luteolin-7-*O*-glucoside. Oleuropein, tyrosol and hydroxytyrosol were quantified, and the results are expressed as mg/100mL as the mean values with ± standard deviation (*n* = 3). The quantification results are presented in Table 5. More information about the percentages of increase or decrease in the compounds’ analytical signals from the target and suspect list that were not quantified (between 3, 6 and 10 min of infusion) can be found in Table S6.

The concentration of oleuropein and hydroxytyrosol increased with the extension of infusion time. The increase in oleuropein concentration was 2.5 times higher from 3 to 6 min of boiling than from 6 to 10 min of boiling. Regarding hydroxytyrosol, its concentration doubled between 3 and 6 min and between 6 and 10 min as well. The levels of concentration of tyrosol were below the LOQ; however, its signal increased with the prolongation of infusion time.

### 3.4. Determination of Bioactive Compounds in Drupes

#### 3.4.1. Target Screening Results for Drupes

From the initial target list of 15 compounds, 7 biophenols were determined in drupes. Those were hydroxytyrosol and tyrosol from the group of phenolic alcohols, the phenolic aldehyde vanillin, apigenin, luteolin and quercetin from the class of flavonoids and the secoiridoid oleuropein. Hydroxytyrosol, tyrosol and vanillin were detected in all drupe varieties except for Koutsourelia from Aetolia-Acarnania-Agrinio and Agouromanakolia from Arcadia-Kynouria. Apigenin was detected in all drupe varieties except for Megaritiki from Attica-Megara and Agouromanakolia from Arcadia-Kynouria. Luteolin was detected in all drupe samples. Quercetin was detected in the varieties Konservolia from Naxos-Melanes, Koutsourelia from Aetolia-Acarnania-Agrinio and Agrilia from Laconia-Sparti. Oleuropein was detected in all drupe varieties except for Agouromanakolia from Arcadia-Kynouria. The target screening results in drupes are summarized in Table 6.

#### 3.4.2. Quantification Results for Drupes

The quantification of oleuropein, tyrosol and hydroxytyrosol in drupes was carried as described in Section 2.6. The quantification results of oleuropein, tyrosol and hydroxytyrosol (expressed in mg/Kg) are presented in Table 7. The concentration of oleuropein in the drupes ranged between below the LOD (Agouromanakolia, Arcadia-Kynouria) and 145 mg/Kg (Throumbolia, Naxos-Melanes). Tyrosol was in the range below the LOD (Agouromanakolia, Arcadia-Kynouria and Koutsourelia, Aetolia-Acarnania-Agrinio) and 40.3 mg/Kg (Koroneiki, Boeotia-Dilesi). Finally, hydroxytyrosol ranged between below the LOD (Agouromanakolia, Arcadia-Kynouria and Koutsourelia, Aetolia-Acarnania-Agrinio) and 426 mg/Kg (Konservolia, Naxos-Melanes).

From the sum of the quantification results, it is concluded that Konservolia from Naxos-Melanes demonstrated the highest phenolic content. Megaritiki from Attica-Megara and Koroneiki from Arcadia-Kynouria also demonstrated higher phenolic content compared to the other varieties.

In drupes, descriptive statistics and statistical analysis were performed for the varieties Koroneiki and Megaritiki (*n* ≥ 3). The quantification results of oleuropein, tyrosol and hydroxytyrosol were examined.

##### Oleuropein

**Figure 10:**
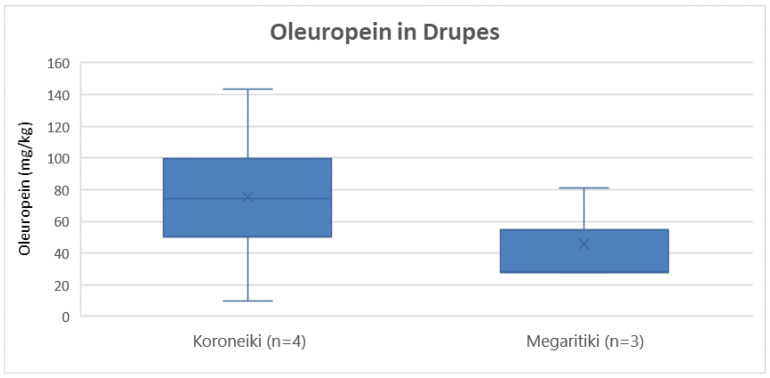
Box and whisker plot for the concentrations of oleuropein between different varieties of drupes.

Comparing the varieties Koroneiki and Megaritiki, Figure 10 shows that the Koroneiki presented higher concentration of oleuropein on average (76 mg/Kg) compared to Megaritiki (46 mg/Kg). However, ANOVA, indicated that there was no statistically significant difference between the varieties (*p* = 0.44).

##### Tyrosol

**Figure 11:**
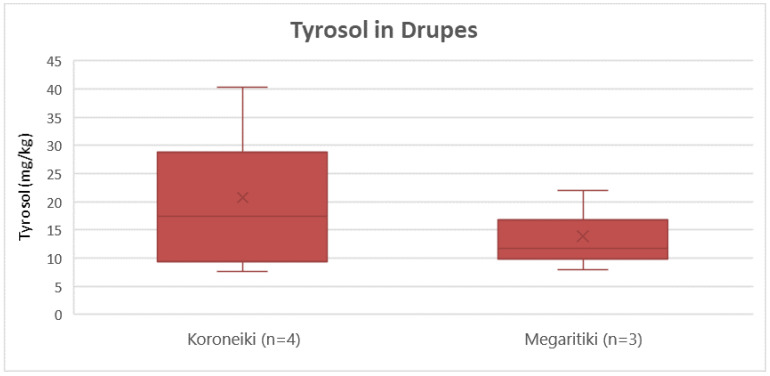
Box and whisker plot for the concentrations of tyrosol between different varieties of drupes.

According to Figure 11, Koroneiki presented a slightly higher concentration of tyrosol on average (21 mg/Kg) compared to Megaritiki (14 mg/Kg). ANOVA showed that this difference was not significant statistically (*p* = 0.51).

##### Hydroxytyrosol

**Figure 12:**
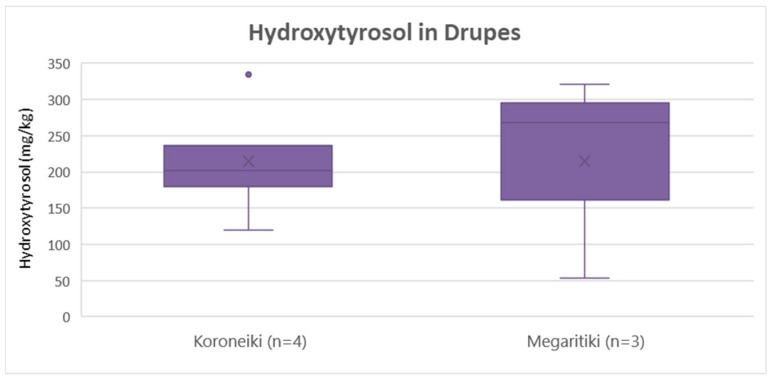
Box and whisker plot for the concentrations of hydroxytyrosol between different varieties of drupes.

As shown in Figure 12, hydroxytyrosol concentration was the same on average (215 mg/Kg) for both Koroneiki and Megaritiki. ANOVA verified that there was no statistically significant difference between the two varieties (*p* = 1.0).

#### 3.4.3. Suspect Screening Results for Drupes

In drupes, 33 bioactive compounds were identified from the initial suspect list of 60 compounds. 4-hydroxyphenylacetic acid, verbascoside, calceolarioside, hydroxytyrosol acetate, hydroxytyrosol glucoside, tyrosol glucoside (or salidroside), apigenin-7-*O*-glucoside, chrysoeriol (or luteolin 3′-methyl ether), diosmin, fustin, luteolin-7-*O*-glucoside, luteolin-7,4′-*O*-diglucoside, quercetin-3-*O*-rutinoside (or rutin), vicenin-2, decarboxymethyl ligstroside aglycone (oleocanthal), decarboxymethyl oleuropein aglycone (oleacein), fraxamoside, ligstroside, ligstroside aglycone, nuzhenide, oleoside, oleuropein aglycone, secologanoside, maslinic acid, oleanolic acid, elenolic acid, elenolic acid 2-*O*-glucoside and suspensaside were identified at level 2a. Demethyl oleuropein, 10-hydroxy-10-methyl oleuropein aglycone, 10-hydroxy decarboxymethyl oleuropein aglycone and 10-hydroxy oleuropein aglycone were identified at level 2b. The presence of vanillic acid was verified with the use of commercial standard.

**Figure 13:**
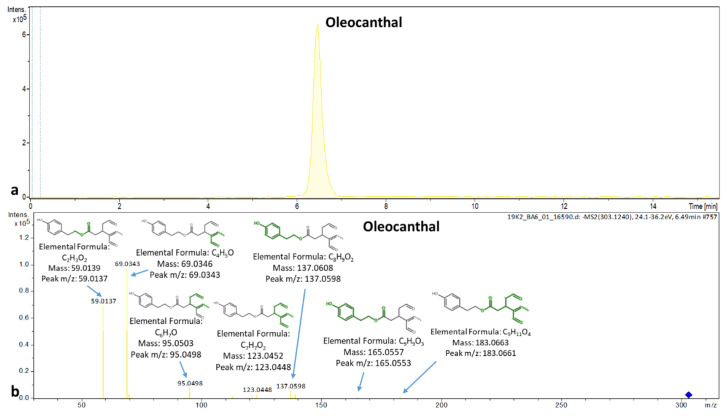
Extracted ion chromatogram and MS/MS spectrum of oleocanthal in drupes.

Furthermore, the compounds nuzhenide, oleocanthal and suspensaside were detected only in the drupes and not in the leaves, whereas the compounds aesculin, the hydroxylated form of elenolic acid, licodione, naringenin, 4-hydroxybenzoic acid and taxifolin were detected only in leaves and not detected in drupes. The EIC and MS/MS spectrum of oleocanthal is presented in Figure 13.

**Figure 14:**
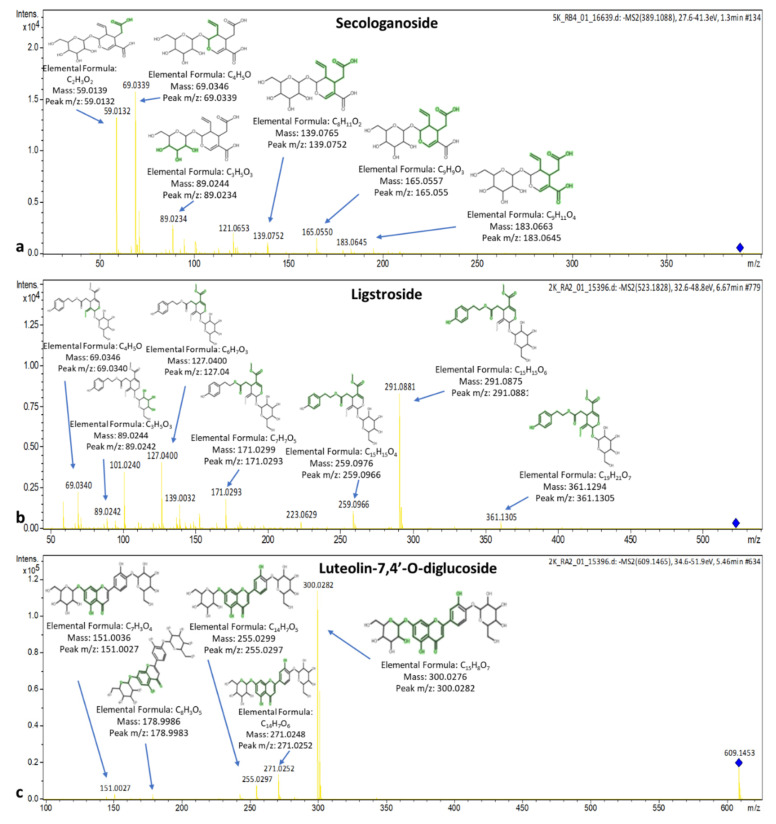
Spectra and MS/MS fragments of (**a**) secologanoside, (**b**) ligstroside, and (**c**) luteolin 7,4′-*O*-diglucoside.

**Figure 15:**
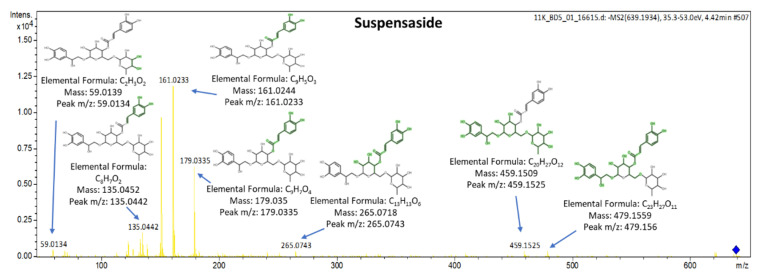
Spectra and MS/MS fragments of suspensaside.

The spectra along with the explained fragments of luteolin-7,4′-*O*-diglucoside (flavonoid), secologanoside (secoiridoid glucoside), and ligstroside are presented in Figure 14. The spectrum of suspensaside (caffeoyl glucoside) is presented in Figure 15. More information about the identification criteria, the level of the identification and the qualifier ions of the suspects can be found in Table S5.

## 4. Conclusions

Τwo novel RP-UHPLC-QTOF-MS/MS methodologies were developed to determine functional ingredients in olive leaves and drupes, respectively. The application of the target screening workflow resulted in the identification of 10 biophenols in leaves and 7 in drupes. Furthermore, 36 suspect compounds were identified in leaves and 33 in drupes with suspect screening. According to the quantification results of oleuropein, tyrosol, hydroxytyrosol and their sum in leaves, the variety Agrilia from Lesvos-Komi exhibited the highest phenolic content, while Kolovi from Lesvos-Palaiohori is ranked second among Koroneiki, Throumbolia, Konservolia, Koutsourelia, Kalamon, Petrolia, Amigdalolia, Megaritiki, Mastoeidis, Agouromakolia and Adramitiani. In the case of drupes, according to the quantification results of the three target compounds, Konservolia from Naxos-Melanes had the highest phenolic content, followed by Megaritiki from Attica-Megara and Koroneiki from Arcadia-Kynouria.

From the class of coumarins, only aesculin was identified in leaves. The triterpenic acids oleanolic and maslinic acid were identified in both matrices. No isochromans were identified with the suggested methods. From lignans, pinoresinol was identified only in leaves of the varieties Petrolia from Serres and Konservolia from Aetolia-Acarnania. Both matrices are rich in secoiridoids. Oleocanthal and nuzhenide were identified only in drupes and not in leaves. The dried olive leaves of Agrilia from Lesvos with the greater phenolic content were dried and infused in boiling water. After the quantification of oleuropein, tyrosol and hydroxytyrosol, it was observed that the optimum infusion time for the preparation of olive leaves tea is 10 min. This work has made progress towards the metabolic fingerprinting of Greek olive leaves and drupes, suggesting that they are a rich source of bioactive constituents.

## Figures and Tables

**Table 1:** Method validation results for leaves.

Compound	LOD (mg/Kg)	LOQ (mg/Kg)	Equation y = (a ± Sa) + (b ± Sb)x (Linear Range: 0.1–8 mg/Kg)	r^2^	Intra-Day Precision RSD_r_ (%) (*n* = 6)	Inter-Day Precision RSD_R_ (%) (*n* × k = 3 × 3)	RE%	ME%
Oleuropein	0.038	0.131	y = (1.18 ± 0.16) + (2.08 ± 0.05)	0.996	5.8	6.5	85.9	−18.6
Tyrosol	0.042	0.125	y = (0.06 ± 0.06) + (1.23 ± 0.02)	0.998	5.4	6.1	93.2	−16.4
Hydroxytyrosol	0.046	0.098	y = (0.18 ± 0.05) + (0.51 ± 0.01)	0.995	4.7	5.8	90.5	−12.7

LOD: limit of detection, LOQ: limit of quantification, r^2^: regression coefficient, RSD: relative standard deviation, RE: recovery, ME: matrix effect.

**Table 2:** Method validation results for drupes.

Compound	LOD (mg/Kg)	LOQ (mg/Kg)	Equation y = (a ± Sa) + (b ± Sb)x (Linear Range: 0.1–8 mg/Kg)	r^2^	Intra-Day Precision RSD_r_, (%) (*n* = 6)	Inter-Day Precision RSD_R_, (%) (*n* × k = 3 × 3)	RE%	ME%
Oleuropein	0.037	0.136	y = (1.15 ± 0.21) + (2.00 ± 0.06)	0.992	5.2	6.2	89.7	−15.4
Tyrosol	0.045	0.098	y = (0.01 ± 0.02) + (1.18 ± 0.01)	0.999	4.6	5.8	92.5	−11.1
Hydroxytyrosol	0.048	0.096	y = (0.13 ± 0.05) + (0.53 ± 0.02)	0.993	3.8	5.1	91.4	−12.8

LOD: limit of detection, LOQ: limit of quantification, r^2^: regression coefficient, RSD: relative standard deviation, RE: recovery, ME: matrix effect.

**Table 3:** Target screening results in leaves.

Compound	Molecular Formula	[M − H]^−^ *m*/*z* Theoretical	[M − H]^−^ *m*/*z* Experimental	t_R_ Standard (min)	Δt_R_ (min)	Fragments *m*/*z*	Elemental Formula
**Phenolic acids**							
Ferulic acid	C_10_H_10_O_4_	193.0506	193.0509	1.40	−0.02	178.0271	C_9_H_6_O_4_
Gallic acid	C_7_H_6_O_5_	169.0142	169.0146	1.25	+0.03	125.0244	C_6_H_5_O_3_
**Phenolic alcohols**							
Hydroxytyrosol	C_8_H_10_O_3_	153.0557	153.0555	3.53	+0.06	123.0444	C_7_H_7_O_2_
Tyrosol	C_8_H_10_O_2_	137.0608	137.0607	4.07	+0.04	81.0267 93.0344 112.0531	C_5_H_5_O C_6_H_5_O C_6_H_8_O_2_
**Phenolic aldehydes**							
Vanillin	C_8_H_8_O_3_	151.0400	151.0401	4.73	−0.03	95.0105 136.0187	C_5_H_3_O_2_ C_7_H_4_O_3_
**Flavonoids**							
Apigenin	C_15_H_10_O_5_	269.0455	269.0454	8.24	−0.01	151.0038	C_7_H_3_O_4_
Luteolin	C_15_H_10_O_6_	285.0404	285.0407	7.46	−0.02	133.0295 151.0036	C_8_H_5_O_2_ C_7_H_3_O_4_
Quercetin	C_15_H_10_O_7_	301.0353	301.0354	7.42	−0.03	121.0293 151.0039	C_7_H_5_O_2_ C_7_H_3_O_4_
**Secoiridoids**							
Oleuropein	C_25_H_32_O_13_	539.1770	539.1770	5.96	−0.02	89.0241 101.0241 307.0821 327.0867 345.0985 377.1258	C_3_H_5_O_3_ C_4_H_5_O_3_ C_15_H_15_O_7_ C_18_H_15_O_6_ C_18_H_17_O_7_ C_19_H_21_O_8_
**Lignans**							
Pinoresinol	C_20_H_22_O_6_	357.1343	357.1346	6.49	+0.04	151.0401	C_8_H_7_O_3_

t_R_: retention time.

**Table 4:** Quantification results of oleuropein, tyrosol and hydroxytyrosol in leaves.

Leaf Sample	Origin	Area	Sample Code	Oleuropein (mg/Kg)	Tyrosol (mg/Kg)	Hydroxytyrosol (mg/Kg)
Koroneiki	Naxos	Melanes	1F	142	47.9	331
Koroneiki	Naxos	Melanes	1F2	163	31.6	391
Throumbolia	Naxos	Melanes	2F	230	15.1	116
Throumbolia	Naxos	Melanes	2F2	199	11.2	74.8
Konservolia	Naxos	Melanes	3F	184	0.625	28.3
Konservolia	Naxos	Melanes	3F2	100	1.30	32.3
Koutsourelia	Aetolia-Acarnania	Agrinio	4F	51.7	18.5	50.2
Koutsourelia	Aetolia-Acarnania	Agrinio	4F2	77.5	26.0	56.4
Konservolia	Aetolia-Acarnania	Agrinio	5F	59.4	4.70	24.2
Konservolia	Aetolia-Acarnania	Agrinio	5F2	35.2	7.01	20.1
Kalamon	Aetolia-Acarnania	Agrinio	6F	41.2	3.77	32.7
Kalamon	Aetolia-Acarnania	Agrinio	6F2	82.9	1.83	27.4
Petrolia	Serres	Skoutari	7F	7.91	<LOD	<LOQ
Petrolia	Serres	Skoutari	7F1	9.45	<LOD	<LOQ
Petrolia	Serres	Skoutari	7F2	1.80	<LOD	<LOQ
Amigdalolia	Attica	Votanikos	8F	114	22.5	211
Amigdalolia	Attica	Votanikos	8F2	60.1	21.2	206
Kalamon	Attica	Votanikos	9F	152	45.4	61.6
Kalamon	Attica	Votanikos	9F2	167	20.0	54.5
Konservolia	Attica	Votanikos	10F	126	9.00	27.1
Konservolia	Attica	Votanikos	10F2	129	2.24	33.9
Koroneiki	Attica	Votanikos	11F	162	3.16	104
Koroneiki	Attica	Votanikos	11F2	115	5.04	112
Koroneiki	Messenia	Kalamata	12F	93.8	29.1	67.9
Kalamon	Messenia	Kalamata	13F	113	9.01	160
Megaritiki	Attica	Aspropyrgos	14F	45.6	20.4	282
Megaritiki	Attica	Sounio	15F	168	5.49	228
Megaritiki	Attica	Megara	16F	200	<LOQ	199
Mastoeidis	Laconia	Sparti	17F	119	8.49	154
Mastoeidis	Laconia	Sparti	17F2	87.2	<LOD	112
Agouromanakolia	Laconia	Sparti	18F	82.0	67.4	240
Agouromanakolia	Laconia	Sparti	18F2	165	68.1	281
Agrilia	Laconia	Sparti	19F	278	34.4	248
Agrilia	Laconia	Sparti	19F2	293	11.1	199
Agouromanakolia	Arcadia	Kynouria	20F	<LOQ	<LOD	<LOD
Megaritiki	Boeotia	Dilesi	21F	5.10	0.373	115
Koroneiki	Arcadia	Kynouria	22F	134	5.42	139
Koroneiki	Boeotia	Dilesi	23F	3.23	19.9	67.4
Agrilia	Lesvos	Komi	24F	212	89.0	421
Agrilia	Lesvos	Komi	24F1	165	114	385
Agrilia	Lesvos	Komi	24F2	149	105	364
Adramitiani	Lesvos	Kalloni	25F	95.0	48.0	102
Adramitiani	Lesvos	Kalloni	25F1	134	35.0	144
Adramitiani	Lesvos	Kalloni	25F2	103	31.0	126
Kolovi	Lesvos	Palaiohori	26F	205	121	378
Kolovi	Lesvos	Palaiohori	26F2	180	97.0	392
Kolovi	Lesvos	Moria	27F	167	112	412

**Table 5:** Quantification results of oleuropein, tyrosol and hydroxytyrosol of olive leaves tea prepared in different infusion times in boiling water.

Infusion Time (min)	Oleuropein (mg/100 mL Olive Leaf Tea ± SD)	Tyrosol (mg/100 mL Olive Leaf Tea)	Hydroxytyrosol (mg/100 mL Olive Leaf Tea ± SD)
3	8.11 ± 0.05	<LOQ	0.06 ± 0.01
6	9.36 ± 0.08	<LOQ	0.11 ± 0.01
10	9.86 ± 0.12	<LOQ	0.27 ± 0.03

**Table 6:** Target screening results in drupes.

Compound	Molecular Formula	[M − H]^−^ *m*/*z* Theoretical	[M − H]^−^ *m*/*z* Experimental		t_R_ Standard (min)	Δt_R_ (min)	Fragments *m*/*z*	Elemental Formula
**Phenolic alcohols**								
Hydroxytyrosol	C_8_H_10_O_3_	153.0557	153.0555	3.52		+0.05	123.0444	C_7_H_7_O_2_
Tyrosol	C_8_H_10_O_2_	137.0608	137.0607	4.07		+0.04	93.0333	C_6_H_5_O
**Phenolic aldehydes**								
Vanillin	C_8_H_8_O_3_	151.0400	151.0401	4.73		−0.03	95.0105 136.0187	C_5_H_3_O_2_ C_7_H_4_O_3_
**Flavonoids**								
Apigenin	C_15_H_10_O_5_	269.0455	269.0454	8.24		−0.01	151.0038	C_7_H_3_O_4_
Luteolin	C_15_H_10_O_6_	285.0404	285.0407	7.46		−0.02	133.0295 151.0036	C_8_H_5_O_2_ C_7_H_3_O_4_
Quercetin	C_15_H_10_O_7_	301.0353	301.0354	7.42		−0.03	121.0293	C_7_H_5_O_2_
**Secoiridoids**								
Oleuropein	C_25_H_32_O_13_	539.1770	539.1770	5.96		−0.02	89.0241 101.0241 307.0821 327.0867 345.0985 377.1258	C_3_H_5_O_3_ C_4_H_5_O_3_ C_15_H_15_O_7_ C_18_H_15_O_6_ C_18_H_17_O_7_ C_19_H_21_O_8_

t_R_: retention time.

**Table 7:** Quantification results of oleuropein, tyrosol and hydroxytyrosol in drupes.

	Origin	Area	Sample No.	Oleuropein (mg/kg)	Tyrosol (mg/kg)	Hydroxytyrosol (mg/kg)
Koroneiki	Naxos	Melanes	1K	143	9.91	203
Throumbolia	Naxos	Melanes	2K	145	7.10	77.8
Konservolia	Naxos	Melanes	3K	96.3	<LOQ	426
Koutsourelia	Aetolia-Acarnania	Agrinio	4K	<LOQ	<LOD	<LOD
Konservolia	Aetolia-Acarnania	Agrinio	5K	90.0	4.72	172
Koroneiki	Attica	Votanikos	11K	85.1	25.0	201
Megaritiki	Attica	Sounio	15K	27.6	7.90	269
Megaritiki	Attica	Megara	16K	81.2	22.0	321
Mastoeidis	Laconia	Sparti	17K	125	19.8	77.8
Agouromanakolia	Laconia	Sparti	18K	133	14.7	78.6
Agrilia	Laconia	Sparti	19K	22.0	7.51	34.0
Agouromanakolia	Arcadia	Kynouria	20K	<LOD	<LOD	<LOD
Megaritiki	Boeotia	Dilesi	21K	28.3	11.7	53.7
Koroneiki	Arkadia	Kynouria	22K	63.8	7.71	335
Koroneiki	Boeotia	Dilesi	23K	10.0	40.3	120

## Supplementary Materials

The following are available online. Table S1: Olive leaves and drupes varieties and geographical origin; Table S2: Quality control results; Table S3: Target list; Table S4: Suspect list; Table S5: Suspect screening results; Table S6: Percentages of increase or decrease in the analytical signals during the experimental time periods of 3 to 6 min and 6 to 10 min.

## References

[1] Morelló J.R., Romero M.P., Ramo T., Motilva M.J. (2005). Evaluation of l-phenylalanine ammonia-lyase activity and phenolic profile in olive drupe (*Olea europaea* L.) from fruit setting period to harvesting time. Plant Sci..

[2] Hashmi M.A., Khan A., Hanif M., Farooq U., Perveen S. (2015). Traditional uses, phytochemistry, and pharmacology of *Olea europaea* (olive). Evidence-Based Complement. Altern. Med..

[3] Kalogeropoulos N., Tsimidou M.Z. (2014). Antioxidants in greek virgin olive oils. Antioxidants.

[4] Erbay Z., Icier F. (2010). The importance and potential uses of olive leaves. Food Rev. Int..

[5] Medfai W., Contreras M.D.M., Lama-Muñoz A., Mhamdi R., Oueslati I., Castro E. (2020). How Cultivar and Extraction Conditions Affect Antioxidants Type and Extractability for Olive Leaves Valorization. ACS Sustain. Chem. Eng..

[6] Japón-Luján R., Luque de Castro M.D. (2007). Small branches of olive tree: A source of biophenols complementary to olive leaves. J. Agric. Food Chem..

[7] Nicolì F., Negro C., Vergine M., Aprile A., Nutricati E., Sabella E., Miceli A., Luvisi A., De Bellis L. (2019). Evaluation of phytochemical and antioxidant properties of 15 Italian *Olea europaea* L. Cultivar Leaves. Molecules.

[8] Bouaziz M., Sayadi S. (2005). Isolation and evaluation of antioxidants from leaves of a Tunisian cultivar olive tree. Eur. J. Lipid Sci. Technol..

[9] Medina E., Romero C., García P., Brenes M. (2019). Characterization of bioactive compounds in commercial olive leaf extracts, and olive leaves and their infusions. Food Funct..

[10] Tsimidou M.Z., Papoti V.T. (2010). Bioactive Ingredients in Olive Leaves.

[11] Japón-Luján R., Janeiro P., De Castro M.D.L. (2008). Solid-liquid transfer of biophenols from olive leaves for the enrichment of edible oils by a dynamic ultrasound-assisted approach. J. Agric. Food Chem..

[12] De Bock M., Thorstensen E.B., Derraik J.G.B., Henderson H.V., Hofman P.L., Cutfield W.S. (2013). Human absorption and metabolism of oleuropein and hydroxytyrosol ingested as olive (*Olea europaea* L.) leaf extract. Mol. Nutr. Food Res..

[13] Michel T., Khlif I., Kanakis P., Termentzi A., Allouche N., Halabalaki M., Skaltsounis A.L. (2015). UHPLC-DAD-FLD and UHPLC-HRMS/MS based metabolic profiling and characterization of different *Olea europaea* organs of Koroneiki and Chetoui varieties. Phytochem. Lett..

[14] Papoti V.T., Papageorgiou M., Dervisi K., Alexopoulos E., Apostolidis K., Petridis D. (2018). Screening olive leaves from unexploited traditional Greek cultivars for their phenolic antioxidant dynamic. Foods.

[15] Kalogiouri N., Samanidou V. (2019). Advances in the Optimization of Chromatographic Conditions for the Separation of Antioxidants in Functional Foods. Rev. Sep. Sci..

[16] Kalogiouri N.P., Aalizadeh R., Dasenaki M.E., Thomaidis N.S. (2020). Application of High Resolution Mass Spectrometric Methods coupled with Chemometric Techniques in Olive Oil Authenticity Studies—A Review. Anal. Chim. ACTA.

[17] Talhaoui N., Gómez-Caravaca A.M., León L., de la Rosa R., Fernández-Gutiérrez A., Segura-Carretero A. (2015). Pattern of Variation of Fruit Traits and Phenol Content in Olive Fruits from Six Different Cultivars. J. Agric. Food Chem..

[18] Santos M.M., Piccirillo C., Castro P.M.L., Kalogerakis N., Pintado M.E. (2012). Bioconversion of oleuropein to hydroxytyrosol by lactic acid bacteria. World J. Microbiol. Biotechnol..

[19] Quirantes-Piné R., Lozano-Sánchez J., Herrero M., Ibáñez E., Segura-Carretero A., Fernández-Gutiérrez A. (2013). HPLC-ESI-QTOF-MS as a powerful analytical tool for characterising phenolic compounds in olive-leaf extracts. Phytochem. Anal..

[20] Kontogianni V.G., Gerothanassis I.P. (2012). Phenolic compounds and antioxidant activity of olive leaf extracts. Nat. Prod. Res..

[21] Talhaoui N., Taamalli A., Gómez-Caravaca A.M., Fernández-Gutiérrez A., Segura-Carretero A. (2015). Phenolic compounds in olive leaves: Analytical determination, biotic and abiotic influence, and health benefits. Food Res. Int..

[22] Kalogiouri N.P., Aalizadeh R., Thomaidis N.S. (2017). Investigating the organic and conventional production type of olive oil with target and suspect screening by LC-QTOF-MS, a novel semi-quantification method using chemical similarity and advanced chemometrics. Anal. Bioanal. Chem..

[23] Kalogiouri N.P., Aalizadeh R., Thomaidis N.S. (2018). Application of an advanced and wide scope non-target screening workflow with LC-ESI-QTOF-MS and chemometrics for the classification of the Greek olive oil varieties. Food Chem..

[24] Kalogiouri N.P., Alygizakis N.A., Aalizadeh R., Thomaidis N.S. (2016). Olive oil authenticity studies by target and nontarget LC–QTOF-MS combined with advanced chemometric techniques. Anal. Bioanal. Chem..

[25] Kalogiouri N.P., Aalizadeh R., Dasenaki M.E., Thomaidis N.S. (2020). Authentication of Greek PDO kalamata table olives: A novel non-target high resolution mass spectrometric approach. Molecules.

[26] Edziri H., Jaziri R., Chehab H., Verschaeve L., Flamini G., Boujnah D., Hammami M., Aouni M., Mastouri M. (2019). A comparative study on chemical composition, antibiofilm and biological activities of leaves extracts of four Tunisian olive cultivars. Heliyon.

[27] Ghasemi S., Koohi D.E., Emmamzadehhashemi M.S.B., Khamas S.S., Moazen M., Hashemi A.K., Amin G., Golfakhrabadi F., Yousefi Z., Yousefbeyk F. (2018). Investigation of phenolic compounds and antioxidant activity of leaves extracts from seventeen cultivars of Iranian olive (*Olea europaea* L.). J. Food Sci. Technol..

[28] Agalias A., Melliou E., Magiatis P., Mitaku S., Gikas E., Tsarbopoulos A. (2005). Quantitation of oleuropein and related metabolites in decoctions of *Olea europaea* leaves from ten greek cultivated varieties by HPLC with diode array detection (HPLC-DAD). J. Liq. Chromatogr. Relat. Technol..

[29] Papoti V.T., Tsimidou M.Z. (2009). Looking through the qualities of a fluorimetric assay for the total phenol content estimation in virgin olive oil, olive fruit or leaf polar extract. Food Chem..

[30] Taamalli A., Arráez-Román D., Zarrouk M., Valverde J., Segura-Carretero A., Fernández-Gutiérrez A. (2012). The Occurrence and Bioactivity of Polyphenols in Tunisian Olive Products and by-Products: A Review. J. Food Sci..

[31] Jemai H., Bouaziz M., Fki I., El Feki A., Sayadi S. (2008). Hypolipidimic and antioxidant activities of oleuropein and its hydrolysis derivative-rich extracts from Chemlali olive leaves. Chem. Biol. Interact..

[32] Kountouri A.M., Mylona A., Kaliora A.C., Andrikopoulos N.K. (2007). Bioavailability of the phenolic compounds of the fruits (drupes) of *Olea europaea* (olives): Impact on plasma antioxidant status in humans. Phytomedicine.

[33] Jemai H., Bouaziz M., Sayadi S. (2009). Phenolic composition, sugar contents and antioxidant activity of tunisian sweet olive cuitivar with regard to fruit ripening. J. Agric. Food Chem..

[34] Bouaziz M., Chamkha M., Sayadi S. (2004). Comparative study on phenolic content and antioxidant activity during maturation of the olive cultivar Chemlali from Tunisia. J. Agric. Food Chem..

[35] Kishikawa A., Ashour A., Zhu Q., Yasuda M., Ishikawa H., Shimizu K. (2015). Multiple biological effects of olive oil by-products such as leaves, stems, flowers, olive milled waste, fruit pulp, and seeds of the olive plant on skin. Phyther. Res..

[36] Khan H., Ahmad W., Hussain I., Imran M., Afridi M.S., Ullah S. (2020). Phytochemical composition, antioxidant and antimicrobial activities of leaves of *Olea europaea* wild variety. J. Food Meas. Charact..

[37] Kontogianni V.G., Charisiadis P., Margianni E., Lamari F.N., Gerothanassis I.P., Tzakos A.G. (2013). Olive leaf extracts are a natural source of advanced glycation end product inhibitors. J. Med. Food.

[38] Ahmad-Qasem M.H., Cánovas J., Barrajón-Catalán E., Micol V., Cárcel J.A., García-Pérez J.V. (2013). Kinetic and compositional study of phenolic extraction from olive leaves (var. Serrana) by using power ultrasound. Innov. Food Sci. Emerg. Technol..

[39] Olmo-García L., Bajoub A., Benlamaalam S., Hurtado-Fernández E., Bagur-González M.G., Chigr M., Mbarki M., Fernández-Gutiérrez A., Carrasco-Pancorbo A. (2018). Establishing the phenolic composition of *Olea europaea* L. Leaves from cultivars grown in Morocco as a crucial step towards their subsequent exploitation. Molecules.

[40] Ryan D., Antolovich M., Herlt T., Prenzler P.D., Lavee S., Robards K. (2002). Identification of phenolic compounds in tissues of the novel olive cultivar Hardy’s Mammoth. J. Agric. Food Chem..

[41] Briante R., Patumi M., Terenziani S., Bismuto E., Febbraio F., Nucci R. (2002). *Olea europaea* L. leaf extract and derivatives: Antioxidant properties. J. Agric. Food Chem..

[42] Goulas V., Papoti V.T., Exarchou V., Tsimidou M.Z., Gerothanassis I.P. (2010). Contribution of flavonoids to the overall radical scavenging activity of olive (*Olea europaea* L.) leaf polar extracts. J. Agric. Food Chem..

[43] Gentile L., Uccella N.A. (2014). Selected bioactives from callus cultures of olives (*Olea europaea* L. Var. Coratina) by LC-MS. Food Res. Int..

[44] Tóth G., Alberti Á., Sólyomváry A., Barabás C., Boldizsár I., Noszál B. (2015). Phenolic profiling of various olive bark-types and leaves: HPLC-ESI/MS study. Ind. Crops Prod..

[45] Giménez E., Juan M.E., Calvo-Melià S., Barbosa J., Sanz-Nebot V., Planas J.M. (2015). Pentacyclic triterpene in *Olea europaea* L.: A simultaneous determination by high-performance liquid chromatography coupled to mass spectrometry. J. Chromatogr. A.

[46] Quirantes-Piné R., Zurek G., Barrajón-Catalán E., Bäßmann C., Micol V., Segura-Carretero A., Fernández-Gutiérrez A. (2013). A metabolite-profiling approach to assess the uptake and metabolism of phenolic compounds from olive leaves in SKBR3 cells by HPLC-ESI-QTOF-MS. J. Pharm. Biomed. Anal..

[47] Jerman Klen T., Golc Wondra A., Vrhovšek U., Mozetič Vodopivec B. (2015). Phenolic Profiling of Olives and Olive Oil Process-Derived Matrices Using UPLC-DAD-ESI-QTOF-HRMS Analysis. J. Agric. Food Chem..

[48] Savarese M., De Marco E., Sacchi R. (2007). Characterization of phenolic extracts from olives (*Olea europaea* cv. Pisciottana) by electrospray ionization mass spectrometry. Food Chem..

[49] Taamalli A., Sánchez J.L., Jebabli H., Trabelsi N., Abaza L., Carretero A.S., Cho J.Y., Román D.A. (2019). Monitoring the bioactive compounds status in *Olea europaea* according to collecting period and drying conditions. Energies.

[50] Kanakis P., Termentzi A., Michel T., Gikas E., Halabalaki M., Skaltsounis A.L. (2013). From olive drupes to olive OilAn HPLC-orbitrap-based qualitative and quantitative exploration of olive key metabolites. Planta Med..

[51] Vinha A.F., Ferreres F., Silva B.M., Valentão P., Gonçalves A., Pereira J.A., Oliveira M.B., Seabra R.M., Andrade P.B. (2005). Phenolic profiles of Portuguese olive fruits (*Olea europaea* L.): Influences of cultivar and geographical origin. Food Chem..

[52] Jimenez P., Masson L., Barriga A., Chávez J., Robert P. (2011). Oxidative stability of oils containing olive leaf extracts obtained by pressure, supercritical and solvent-extraction. Eur. J. Lipid Sci. Technol..

[53] El S.N., Karakaya S. (2009). Olive tree (*Olea europaea*) leaves: Potential beneficial effects on human health. Nutr. Rev..

[54] Kiritsakis K., Kontominas M.G., Kontogiorgis C., Hadjipavlou-Litina D., Moustakas A., Kiritsakis A. (2010). Composition and antioxidant activity of olive leaf extracts from Greek olive cultivars. JAOCS J. Am. Oil Chem. Soc..

[55] Cittan M., Çelik A. (2018). Development and validation of an analytical methodology based on liquid chromatography-electrospray tandem mass spectrometry for the simultaneous determination of phenolic compounds in olive leaf extract. J. Chromatogr. Sci..

[56] Capriotti A.L., Cavaliere C., Crescenzi C., Foglia P., Nescatelli R., Samperi R., Laganà A. (2014). Comparison of extraction methods for the identification and quantification of polyphenols in virgin olive oil by ultra-HPLC-QToF mass spectrometry. Food Chem..

[57] Gago-Ferrero P., Schymanski E.L., Bletsou A.A., Aalizadeh R., Hollender J., Thomaidis N.S. (2015). Extended Suspect and Non-Target Strategies to Characterize Emerging Polar Organic Contaminants in Raw Wastewater with LC-HRMS/MS. Environ. Sci. Technol..

[58] Wolf S., Schmidt S., Müller-Hannemann M., Neumann S. (2010). In silico fragmentation for computer assisted identification of metabolite mass spectra. BMC Bioinform..

[59] Aalizadeh R., Thomaidis N.S., Bletsou A.A., Gago-Ferrero P. (2016). Quantitative Structure-Retention Relationship Models to Support Nontarget High-Resolution Mass Spectrometric Screening of Emerging Contaminants in Environmental Samples. J. Chem. Inf. Model..

[60] Schymanski E.L., Jeon J., Gulde R., Fenner K., Ruff M., Singer H.P., Hollender J. (2014). Identifying small molecules via high resolution mass spectrometry: Communicating confidence. Environ. Sci. Technol..

