# Supplementary material for: Target and Suspect HRMS Metabolomics for the Determination of Functional Ingredients in 13 Varieties of Olive Leaves and Drupes from Greece
Source: Molecules. 2020 Oct 22;25(21):4889. doi: 10.3390/molecules25214889 (PMC7660111; doi:10.3390/molecules25214889)
Supplement: Supplementary file 1 [file molecules-25-04889-s001.pdf]

molecules

SUPPLEMENTARY MATERIAL

Target and suspect HRMS metabolomics for the determination of functional ingredients in 13 varieties of olive leaves and drupes from Greece

Evangelia Kritikou, Natasa P. Kalogiouri, Lydia Kolyvira and Nikolaos S. Thomaidis*

Laboratory of Analytical Chemistry, Department of Chemistry, National and Kapodistrian University of Athens, Panepistimiopolis Zographou, 15771 Athens, Greece
* Correspondence: ntho@chem.uoa.gr

Received: date; Accepted: date; Published: date

Table S1. Olive leaves and drupes varieties and geographical origin

Variety	Geographical Origin	Sample	
		**Leaf**	**Drupe**
		Sample no	*Sample no*
Koroneiki	Naxos (Melanes)	1F, 1F2	1K
Throumbolia	Naxos (Melanes)	2F, 2F2	2K
Konservolia	Naxos (Melanes)	3F, 3F2	3K
Koutsourelia	Aetolia-Acarnania (Agrinio)	4F, 4F2	4K
Konservolia	Aetolia-Acarnania (Agrinio)	5F, 5F2	5K
Kalamon	Aetolia-Acarnania (Agrinio)	6F, 6F2	-
Petrolia	Serres (Skoutari)	7F, 7F1, 7F2	-
Amigdalolia	Attica (Votanikos)	8F, 8F2	-
Kalamon	Attica (Votanikos)	9F, 9F2	-
Konservolia	Attica (Votanikos)	10F, 10F2	-
Koroneiki	Attica (Votanikos)	11F, 11F2	11K
Koroneiki	Messenia (Kalamata)	12F	-
Kalamon	Messenia (Kalamata)	13F	-
Megaritiki	Attica (Aspropyrgos)	14F	-
Megaritiki	Attica (Sounio)	15F	15K
Megaritiki	Attica (Megara)	16F	16K
Mastoeidis	Laconia (Sparti)	17F, 17F2	17K
Agouromanakolia	Laconia (Sparti)	18F, 18F2	18K
Agrilia	Laconia (Sparti)	19F, 19F2	19K
Agouromanakolia	Arcadia (Kynouria)	20F	20K
Megaritiki	Boeotia (Dilesi)	21F	21K
Koroneiki	Arcadia (Kynouria)	22F	22K
Koroneiki	Boeotia (Dilesi)	23F	23K
Agrilia	Lesvos (Komi)	24F, 24F1, 24F2	-
Adramitiani	Lesvos (Kalloni)	25F, 25F1, 25F2	-
Kolovi	Lesvos (Palaiohori)	26F, 26F2	-
Kolovi	Lesvos (Moria)	27F	-

Table S2. Quality Control results

Compound	%RSD of Peak Area n=11	%RSD of t_R (min) n=11	Δm (±error, mDa) n=11
Oleuropein	0.53	0.05	-0.08
Tyrosol	0.48	0.05	0.03
Hydroxytyrosol	0.61	0.06	0.10

Table S3. Target list

**Compound**	**Molecular Formula**
**Phenolic acids**	
Caffeic acid	$C_9H_8O_4$
Ferulic acid	$C_{10}H_{10}O_4$
Gallic acid	$C_7H_6O_5$
Homovanillic acid	$C_9H_{10}O_4$
p-Coumaric acid	$C_9H_8O_3$
Syringic acid	$C_9H_{10}O_5$
**Phenolic alcohols**	
Hydroxytyrosol	$C_8H_{10}O_3$
Tyrosol	$C_8H_{10}O_2$
**Phenolic aldehydes**	
Vanillin	$C_8H_8O_3$
**Flavonoids**	
Apigenin	$C_{15}H_{10}O_5$
Epicatechin	$C_{15}H_{14}O_6$
Luteolin	$C_{15}H_{10}O_6$
Quercetin	$C_{15}H_{10}O_7$
**Secoiridoids**	
Oleuropein	$C_{25}H_{32}O_{13}$
**Lignans**	
Pinoresinol	$C_{20}H_{22}O_6$

Table S4. Suspect list

Compound	Molecular Formula	SMILES
**Phenolic acids and derivatives**		
2,5-Dihydroxybenzoic acid (Gentisic acid)	$C_7H_6O_4$	OC(=O)C1=CC(O)=CC=C1O
3,4-Dihydroxybenzoic acid (Protocatechuic acid)	$C_7H_6O_4$	OC(=O)C1=CC(O)=C(O)C=C1
Hellicoside	$C_{29}H_{36}O_{17}$	c1cc(c(cc1/C=C/C(=O)O[C@@H]2[C@H](O[C@H]([C@@H]([C@H]2O[C@H]3[C@@H]([C@H]([C@@H]([C@H](O3)CO)O)O)O)O)OC[C@H](c4ccc(c(c4)O)O)O)CO)O)O
4-Hydroxybenzoic acid	$C_7H_6O_3$	OC(=O)C1=CC=C(O)C=C1
4-Hydroxyphenylacetic acid	$C_8H_8O_3$	OC(C(O)=O)C1=CC=CC=C1
Rosmarinic acid	$C_{18}H_{16}O_8$	c1cc(c(cc1C[C@H](C(=O)O)OC(=O)/C=C/c2ccc(c(c2)O)O)O)O
Vanillic acid	$C_8H_8O_4$	COc1cc(ccc1O)C(=O)O
Verbascoside	$C_{29}H_{36}O_{15}$	CC1OC(OC2C(O)C(OCCC3=CC=C(O)C(O)=C3)OC(CO)C2OC(=O)C=CC2=CC=C(O)C(O)=C2)C(O)C(O)C1O
**Phenolic alcohols and derivatives**		
Calceolarioside	$C_{23}H_{26}O_{11}$	OC[C@H]1O[C@@H](OCCC2=CC=C(O)C(O)=C2)[C@H](O)[C@@H](O)[C@@H]1OC(=O)\C=C\C1=CC(O)=C(O)C=C1
Homovanillyl alcohol	$C_9H_{12}O_3$	COC1=CC(CCO)=CC=C1O
Hydroxytyrosol acetate	$C_{10}H_{12}O_4$	CC(=O)OCCC1=CC(O)=C(O)C=C1
Hydroxytyrosol glucoside	$C_{14}H_{20}O_8$	OC[C@H]1O[C@@H](OCCC2=CC(O)=C(O)C=C2)[C@H](O)[C@@H](O)[C@@H]1O
Tyrosol glucoside (Salidroside)	$C_{14}H_{20}O_7$	[H][C@]1(CO)O[C@@]([H])(OCCC2=CC=C(O)C=C2)[C@]([H])(O)[C@@]([H])(O)[C@]1([H])O

**Compound**	**Molecular Formula**	**SMILES**
		**Flavonoids**
Apigenin-7-O-glucoside	$C_{21}H_{20}O_{10}$	OC[C@H]1O[C@@H](OC2=CC(O)=C3C(=O)C=C(OC3=C2)C2=CC=C(O)C=C2)[C@H](O)[C@@H](O)[C@@H]1O
Chrysoeriol (Luteolin 3'-methyl ether)	$C_{16}H_{12}O_6$	COC1=CC(=CC=C1O)C1=CC(=O)C2=C(O)C=C(O)C=C2O1
Diosmetin	$C_{16}H_{12}O_6$	COC1=CC=C(C=C1O)C1=CC(=O)C2=C(O)C=C(O)C=C2O1
Diosmin	$C_{28}H_{32}O_{15}$	COC1=CC=C(C=C1O)C1=CC(=O)C2=C(O)C=C(O[C@@H]3O[C@H](CO[C@@H]4O[C@@H](C)[C@H](O)[C@@H](O)[C@H]4O)[C@@H](O)[C@H](O)[C@H]3O)C=C2O1
Fustin	$C_{15}H_{12}O_6$	c1cc(c(cc1[C@@H]2[C@H](C(=O)c3ccc(cc3O2)O)O)O)O
Gallocatechin	$C_{15}H_{14}O_7$	O[C@@H]1CC2=C(O)C=C(O)C=C2O[C@H]1C1=CC(O)=C(O)C(O)=C1
Luteolin-7-O-glucoside	$C_{21}H_{20}O_{11}$	OC[C@@H](O)[C@H]1O[C@@H](OC2=C(OC3=CC(O)=CC(O)=C3C2=O)C2=CC(O)=C(O)C=C2)[C@H](O)[C@H]1O
Luteolin-7,4′-O-diglucoside	$C_{27}H_{30}O_{16}$	OC[C@@H]1O[C@@H](OC2=CC(O)=C3C(=O)C=C(OC3=C2)C2=CC(O[C@@H]3O[C@@H](CO)[C@H](O)[C@@H](O)[C@@H]3O)=C(O)C=C2)[C@@H](O)[C@H](O)[C@H]1O
Naringenin	$C_{15}H_{12}O_5$	c1cc(ccc1[C@@H]2CC(=O)c3c(cc(cc3O2)O)O)O
Quercetin-3-O-glucoside	$C_{21}H_{20}O_{12}$	OC[C@@H](O)[C@H]1O[C@@H](OC2=C(OC3=CC(O)=CC(O)=C3C2=O)C2=CC(O)=C(O)C=C2)[C@H](O)[C@H]1O
Quercetin-3-O-rutinoside (Rutin)	$C_{27}H_{30}O_{16}$	CC1OC(OCC2OC(OC3=C(OC4=CC(O)=CC(O)=C4C3=O)C3=CC=C(O)C(O)=C3)C(O)C(O)C2O)C(O)C(O)C1O
Taxifolin	$C_{15}H_{12}O_7$	OC1C(OC2=CC(O)=CC(O)=C2C1=O)C1=CC=C(O)C(O)=C1

Compound	Molecular Formula	SMILES
Vicenin-2	$C_{27}H_{30}O_{15}$	c1cc(ccc1c2cc(=O)c3c(c(c(c(c3o2)[C@H]4[C@@H]([C@H]([C@@H]([C@H](O4)CO)O)O)O)O)[C@H]5[C@@H]([C@H]([C@@H]([C@H](O5)CO)O)O)O)O)O
	**Secoiridoids**	
Decarboxymethyl ligstroside aglycone (Oleocanthal)	$C_{17}H_{20}O_5$	C\C=C(\C=O)[C@@H](CC=O)CC(=O)OCCC1=CC=C(O)C=C1
Decarboxymethyl oleuropein aglycone (Oleacein)	$C_{17}H_{20}O_6$	C\C=C(\C=O)[C@@H](CC=O)CC(=O)OCCC1=CC(O)=C(O)C=C1
Demethyl oleuropein	$C_{24}H_{30}O_{13}$	C\C=C1\[C@H](O[C@@H]2O[C@H](CO)[C@@H](O)[C@H](O)[C@H]2O)OC=C([C@H]1CC(=O)OCCC1=CC=C(O)C(O)=C1)C(O)=O
Fraxamoside	$C_{25}H_{30}O_{13}$	[H][C@@]12CC(=O)OC[C@H](OC[C@H]3O[C@@H](O[C@H](OC=C1C(=O)OC)\C2=C\C)[C@H](O)[C@@H](O)[C@@H]3O)C1=CC(O)=C(O)C=C1
10-Hydroxy-10-Methyl oleuropein aglycone	$C_{20}H_{24}O_9$	COC(=O)C1=CO[C@@H](O)\C(=C/C(C)O)[C@@H]1CC(=O)OCCC1=CC(O)=C(O)C=C1
10-Hydroxy decarboxymethyl oleuropein aglycone	$C_{17}H_{20}O_7$	[H]C(O)\C=C1\C(O)OC=C[C@H]1CC(=O)OCCC1=CC(O)=C(O
10-Hydroxy oleuropein aglycone	$C_{19}H_{22}O_9$	[H]C(O)\C=C1/[C@H](O)OC=C([C@H]1CC(=O)OCCC1=CC(O)=C(O)C=C1)C(=O)OC
Ligstroside	$C_{25}H_{32}O_{12}$	CC1OC(OC2C(O)C(OCCC3=CC=C(O)C(O)=C3)OC(CO)C2OC(=O)C=CC2=CC=C(O)C(O)=C2)C(O)C(O)C1O
Ligstroside aglycone	$C_{19}H_{22}O_7$	COC(=O)C1=CO[C@@H](O)\C(=C\C)[C@@H]1CC(=O)OCCC1=CC=C(O)C=C1
Nuzhenide	$C_{31}H_{42}O_{17}$	COC(=O)C1=CO[C@@H](O[C@@H]2O[C@H](CO)[C@@H](O)[C@H](O)[C@H]2O)\C(=C/C)C1CC(=O)OC[C@@H]1O[C@H](OCCC2=CC=C(O)C=C2)[C@@H](O)[C@H](O)[C@H]1O

Compound	Molecular Formula	SMILES
Oleoside	$C_{16}H_{22}O_{11}$	[H][C@]1(CO)O[C@@]([H])(O[C@]2([H])OC=C(C(O)=O)[C@@]([H])(CC(O)=O)\C2=C/C)[C@]([H])(O)[C@@]([H])(O)[C@]1([H])O
Oleuropein aglycone	$C_{19}H_{22}O_8$	COC(=O)C1=CO[C@@H](O)\C(=C\C)[C@@H]1CC(=O)OCCC1=CC(O)=C(O)C=C1
Secologanoside	$C_{16}H_{22}O_{11}$	OC[C@H]1O[C@@H](O[C@@H]2OC=C([C@@H](CC(O)=O)[C@H]2C=C)C(O)=O)[C@H](O)[C@@H](O)[C@@H]1O
**Lignans**		
Acetoxypinoresinol	$C_{22}H_{24}O_8$	COC1=C(O)C=CC(=C1)C1OCC2(OC(C)=O)C1COC2C1=CC(OC)=C(O)C=C1
Berchemol	$C_{20}H_{24}O_7$	COC1=C(C=CC(=C1)CC2COC(C2(CO)O)C3=CC(=C(C=C3)O)OC)O
Hydroxypinoresinol	$C_{20}H_{22}O_7$	COC1=C(O)C=CC(=C1)C1OCC2(O)C1COC2C1=CC(OC)=C(O)C=C1
Olivil	$C_{20}H_{24}O_7$	COC1=C(O)C=CC(C[C@@]2(O)CO[C@@H]([C@H]2CO)C2=CC(OC)=C(O)C=C2)=C1
Syringaresinol	$C_{22}H_{26}O_8$	COC1=CC(=CC(OC)=C1O)C1OCC2C1COC2C1=CC(OC)=C(O)C(OC)=C1
**Hydroxy-isochromans**		
1-(3′-methoxy-4′-hydroxy)-phenyl-6,7-dihydroxy-isochroman	$C_{16}H_{16}O_5$	COC1=C(O)C=CC(=C1)C1=C(O)C(O)=CC2=C1COCC2
1-phenyl-6,7-dihydroxy-isochroman	$C_{15}H_{14}O_3$	OC1=C(O)C=C2C(OCCC2=C1)C1=CC=CC=C1
**Triterpenic acids**		
Maslinic acid	$C_{30}H_{48}O_4$	CC1(C)CC[C@@]2(CC[C@]3(C)C(=CC[C@@H]4[C@@]5(C)C[C@@H](O)[C@H](O)C(C)(C)[C@@H]5CC[C@@]34C)[C@@H]2C1)C(O)=O
Oleanolic acid	$C_{30}H_{48}O_3$	CC1(C)CC[C@@]2(CC[C@]3(C)C(=CC[C@@H]4[C@@]5(C)CC[C@H](O)C(C)(C)[C@@H]5CC[C@@]34C)[C@@H]2C1)C(O)=O
**Coumarins**		

**Compound**	**Molecular Formula**	**SMILES**
Aesculin	$C_{15}H_{15}O_9$	OC[C@H]1O[C@@H](OC2=C(O)C=C3OC(=O)C=CC3=C2)[C@H](O)[C@@H](O)[C@@H]1O
Cichoriin	$C_{15}H_{16}O_9$	C1=CC(=O)OC2=CC(=C(C=C21)O)OC3C(C(C(C(O3)CO)O)O)O
Esculetin	$C_9H_6O_4$	C1=CC(=O)OC2=CC(=C(C=C21)O)O
Scopoletin	$C_{10}H_8O_4$	COC1=C(C=C2C(=C1)C=CC(=O)O2)O
**Other compounds**		
Abscisic acid	$C_{15}H_{20}O_4$	C\C(\C=C\[C@@]1(O)C(C)=CC(=O)CC1(C)C)=C\C(O)=O
Azelaic acid	$C_9H_{16}O_4$	OC(=O)CCCCCCCC(O)=O
Elenolic acid	$C_{11}H_{14}O_6$	COC(=O)C1=CO[C@@H](C)[C@@H](C=O)[C@@H]1CC(O)=O
Elenolic acid 2-O-glucoside (Oleoside 11-methyl ester)	$C_{17}H_{24}O_{11}$	COC(=O)C1=CO[C@@H](O[C@@H]2O[C@H](CO)[C@@H](O)[C@H](O)[C@H]2O)\C(=C\C)[C@@H]1CC(O)=O
Elenolic acid methyl ester	$C_{12}H_{16}O_6$	COC(=O)C[C@H]1[C@H](C=O)[C@H](C)OC=C1C(=O)OC
Hydroxylated form of elenolic acid	$C_{11}H_{14}O_7$	COC(=O)C1=CO[C@@H](CO)[C@@H](C=O)[C@@H]1CC(O)=O
Licodione	$C_{15}H_{12}O_5$	OC1=CC=C(C=C1)C(=O)CC(=O)C1=CC=C(O)C=C1O
Suspensaside	$C_{29}H_{36}O_{16}$	CC1C(C(C(C(O1)OCC2C(C(C(C(O2)OCC(C3=CC(=C(C=C3)O)O)O)O)O)OC(=O)C=CC4=CC(=C(C=C4)O)O)O)O)O

SMILES: simplified molecular-input line-entry system

Table S5. Suspect screening results

Compound	Molecular Formula	[M-H]- m/z calc.	[M-H]- m/z exp.	t_R exp. (min)	t_R QSRR (min)	Fragments m/z	Elemental Formula	Peak score A/I	Leaf	Drupe	Level Ident.
**Phenolic acids and derivatives**											
4-Hydroxybenzoic acid	$C_7H_6O_3$	137.0244	137.0243	4.00	4.45	71.0139<br>108.0217<br>123.0088	$C_3H_3O_2$<br>$C_6H_4O_2$<br>$C_6H_3O_3$	16	Detected	-	2a
4-Hydroxyphenylacetic acid	$C_8H_8O_3$	151.0400	151.0402	3.44	4.00	71.0135<br>81.0341<br>95.0499<br>108.0215<br>123.0448<br>137.0238	$C_3H_3O_2$<br>C_5H_5O<br>C_6H_7O<br>$C_6H_4O_2$<br>$C_7H_7O_2$<br>$C_7H_5O_3$	13	Detected	Detected	2a
Vanillic acid	$C_8H_8O_4$	167.0349	167.0344	1.43	-	71.0131<br>93.0364<br>123.0454<br>137.0257<br>149.0209	$C_3H_3O_2$<br>C_6H_5O<br>$C_7H_7O_2$<br>$C_7H_5O_3$<br>$C_8H_5O_3$	11	Detected	Detected	1
Verbascoside	$C_{29}H_{36}O_{15}$	623.1981	623.1983	5.00	6.91	113.0242<br>133.0292<br>153.0566<br>161.0250<br>179.0351<br>241.0714<br>275.0569<br>315.1109<br>461.1676	$C_5H_5O_3$<br>$C_8H_5O_2$<br>$C_8H_9O_3$<br>$C_9H_5O_3$<br>$C_9H_7O_4$<br>$C_{11}H_{13}O_6$<br>$C_{14}H_{11}O_6$<br>$C_{14}H_{19}O_8$<br>$C_{20}H_{29}O_{12}$	7	Detected	Detected	2a
**Phenolic alcohols and derivatives**											

**Compound**	**Molecular Formula**	**$[M-H]^-$ m/z calc.**	**$[M-H]^-$ m/z exp.**	**t_R exp. (min)**	**t_R QSRR (min)**	**Fragments m/z**	**Elemental Formula**	**Peak score A/I**	**Leaf**	**Drupe**	**Level Ident.**
Calceolarioside	$C_{23}H_{26}O_{11}$	477.1402	477.1406	5.69	7.64	217.0502<br>285.0977	$C_{12}H_9O_4$<br>$C_{13}H_{17}O_7$	8	Detected	Detected	2a
Hydroxytyrosol acetate	$C_{10}H_{12}O_4$	195.0660	195.0664	6.65	6.48	59.0139<br>121.0295<br>149.0608	$C_2H_3O_2$<br>$C_7H_5O_2$<br>$C_9H_9O_2$	13	Detected	Detected	2a
Hydroxytyrosol glucoside	$C_{14}H_{20}O_8$	315.1085	315.1085	3.88	4.15	71.0132<br>105.0337<br>113.0228<br>119.0337<br>123.0447<br>135.0446<br>153.0549<br>179.0578<br>240.0601	$C_3H_3O_2$<br>C_7H_5O<br>$C_5H_5O_3$<br>$C_3H_8O_4$<br>$C_7H_7O_2$<br>$C_8H_7O_2$<br>$C_8H_9O_3$<br>$C_6H_{11}O_6$<br>$C_{11}H_{13}O_6$	13	Detected	Detected	2a
Tyrosol glucoside (Salidroside)	$C_{14}H_{20}O_7$	299.1136	299.1135	3.94	4.92	71.0134<br>113.0222<br>137.0599	$C_3H_3O_2$<br>$C_5H_5O_3$<br>$C_8H_9O_2$	9	Detected	Detected	2a
					**Flavonoids**						
Apigenin-7-O-glucoside	$C_{21}H_{20}O_{10}$	431.0983	431.0987	5.92	5.38	67.0188<br>111.0086<br>143.0353<br>151.0037<br>269.0444<br>300.0276<br>311.0555	C_4H_3O<br>$C_5H_3O_3$<br>$C_6H_7O_4$<br>$C_8H_5O_3$<br>$C_{15}H_9O_5$<br>$C_{15}H_8O_7$<br>$C_{17}H_1O_6$	7	Detected	Detected	2a

**Compound**	**Molecular Formula**	**[M-H]⁻ m/z calc.**	**[M-H]⁻ m/z exp.**	**t_R exp. (min)**	**t_R QSRR (min)**	**Fragments m/z**	**Elemental Formula**	**Peak score A/I**	**Leaf**	**Drupe**	**Level Ident.**
Chrysoeriol (Luteolin 3'-methyl ether)	$C_{16}H_{12}O_6$	299.0561	299.0560	8.24	7.41	83.0138<br>107.0139<br>134.0352<br>151.0024<br>158.0369<br>200.0464<br>227.0345<br>256.0376<br>284.0327	$C_4H_3O_2$<br>$C_6H_3O_2$<br>$C_8H_6O_2$<br>$C_7H_3O_4$<br>$C_{10}H_6O_2$<br>$C_{12}H_8O_3$<br>$C_{13}H_7O_4$<br>$C_{14}H_8O_5$<br>$C_{15}H_8O_6$	10	Detected	Detected	2a
Diosmin	$C_{28}H_{32}O_{15}$	607.1668	607.1669	5.70	6.00	89.0242<br>151.0401<br>162.0524<br>171.0296<br>216.0404<br>233.0664<br>256.0374<br>275.0768<br>284.0307<br>299.0537<br>301.0351<br>307.1014<br>343.0448<br>397.1134<br>444.1261	$C_3H_5O_3$<br>$C_8H_7O_3$<br>$C_6H_{10}O_5$<br>$C_7H_7O_5$<br>$C_{12}H_8O_4$<br>$C_9H_{13}O_7$<br>$C_{14}H_8O_5$<br>$C_{11}H_{15}O_8$<br>$C_{15}H_8O_6$<br>$C_{16}H_{11}O_6$<br>$C_{15}H_9O_7$<br>$C_{12}H_{19}O_9$<br>$C_{17}H_{11}O_8$<br>$C_{18}H_{21}O_{10}$<br>$C_{19}H_{24}O_{12}$	5	Detected	Detected	2a

Compound	Molecular Formula	[M-H]- m/z calc.	[M-H]- m/z exp.	t_R exp. (min)	t_R QSRR (min)	Fragments m/z	Elemental Formula	Peak score A/I	Leaf	Drupe	Level Ident.
Fustin	$C_{15}H_{12}O_6$	287.0561	287.0560	6.27	5.77	83.0133	$C_4H_3O_2$	9	Detected	Detected	2a
						107.0137	$C_6H_3O_2$				
						125.0240	$C_6H_5O_3$				
						135.0446	$C_8H_7O_2$				
						151.0031	$C_7H_3O_4$				
						161.0236	$C_9H_5O_3$				
						169.0133	$C_7H_5O_5$				
						177.0181	$C_9H_5O_4$				
						185.0252	$C_{11}H_5O_3$				
						203.0352	$C_{11}H_7O_4$				
						269.0462	$C_{15}H_9O_5$				
Luteolin-7-O-glucoside	$C_{21}H_{20}O_{11}$	447.0932	447.0933	5.41	5.07	59.0135	$C_2H_3O_2$	16	Detected	Detected	2a
						133.0289	$C_8H_5O_2$				
						151.0033	$C_7H_3O_4$				
						169.0137	$C_7H_5O_5$				
						211.0387	$C_{13}H_7O_3$				
						227.0330	$C_{13}H_7O_4$				
						256.0386	$C_{14}H_8O_5$				
						285.0399	$C_{15}H_9O_6$				
						327.0497	$C_{17}H_{11}O_7$				
Luteolin-7,4′-O-diglucoside	$C_{27}H_{30}O_{16}$	609.1461	609.1463	5.45	5.14	113.0233	$C_5H_5O_3$	12	Detected	Detected	2a
						151.0027	$C_7H_3O_4$				
						178.9983	$C_8H_3O_5$				
						255.0297	$C_{14}H_7O_5$				
						271.0252	$C_{14}H_7O_6$				
						300.0282	$C_{15}H_8O_7$				
						343.0469	$C_{17}H_{11}O_8$				

Compound	Molecular Formula	$[M-H]^-$ m/z calc.	$[M-H]^-$ m/z exp.	t_R exp. (min)	t_R QSRR (min)	Fragments m/z	Elemental Formula	Peak score A/I	Leaf	Drupe	Level Ident.
						591.1407	$C_{27}H_{27}O_{15}$				
Naringenin	$C_{15}H_{12}O_5$	271.0611	271.0616	7.41	7.48	119.0504	C_8H_7O	6	Detected	-	2a
						145.0294	$C_9H_5O_2$				
						173.0242	$C_{10}H_5O_3$				
						177.0163	$C_9H_5O_4$				
						187.0369	$C_{11}H_7O_3$				
Quercetin-3-O-rutinoside (Rutin)	$C_{27}H_{30}O_{16}$	609.1461	609.1465	4.61	4.26	113.0233	$C_5H_5O_3$	12	Detected	Detected	2a
						151.0027	$C_7H_3O_4$				
						161.0435	$C_6H_9O_5$				
						272.0300	$C_{14}H_8O_6$				
						300.0282	$C_{15}H_8O_7$				
						343.0469	$C_{17}H_{11}O_8$				
						447.0923	$C_{21}H_{19}O_{11}$				
Taxifolin	$C_{15}H_{12}O_7$	303.0510	303.0517	4.86	-	83.0136	$C_4H_3O_2$	5	Detected	-	1
						123.0445	$C_7H_7O_2$				
						125.0245	$C_6H_5O_3$				
						137.0232	$C_7H_5O_3$				
						151.0047	$C_7H_3O_4$				
						175.0393	$C_{10}H_7O_3$				
						193.0513	$C_{10}H_9O_4$				
						217.0515	$C_{12}H_9O_4$				
						285.0450	$C_{15}H_9O_6$				
Vicenin-2	$C_{27}H_{30}O_{15}$	593.1511	593.1518	4.12	3.92	151.0396	$C_8H_7O_3$	6	Detected	Detected	2a
						325.0698	$C_{18}H_{13}O_6$				
						353.0661	$C_{19}H_{13}O_7$				
						383.0755	$C_{20}H_{15}O_8$				

Compound	Molecular Formula	[M-H]⁻ m/z calc.	[M-H]⁻ m/z exp.	t_R exp. (min)	t_R QSRR (min)	Fragments m/z	Elemental Formula	Peak score A/I	Leaf	Drupe	Level Ident.
						413.0889<br>473.1113<br>503.1198<br>575.1431	$C_{21}H_{17}O_9$<br>$C_{23}H_{21}O_{11}$<br>$C_{24}H_{23}O_{12}$<br>$C_{27}H_{27}O_{14}$				
					**Secoiridoids**						
Decarboxymethyl ligstroside aglycone (Oleocanthal)	$C_{17}H_{20}O_5$	303.1237	303.1239	6.47	6.76	69.0343<br>95.0498<br>113.0235<br>121.0294<br>123.0448<br>137.0598<br>165.0553<br>183.0661<br>233.0819	C_4H_5O<br>C_6H_7O<br>$C_5H_5O_3$<br>$C_7H_5O_2$<br>$C_7H_7O_2$<br>$C_8H_9O_2$<br>$C_9H_9O_3$<br>$C_9H_{11}O_4$<br>$C_{13}H_{13}O_4$	4	-	Detected	2a
Decarboxymethyl oleuropein aglycone (Oleacein)	$C_{17}H_{20}O_6$	319.1185	319.1188	5.69	6.14	69.0346<br>95.0499<br>113.0227<br>123.0448<br>139.0390<br>139.0757<br>165.0541<br>183.0658	C_4H_5O<br>C_6H_7O<br>$C_5H_5O_3$<br>$C_7H_7O_2$<br>$C_7H_7O_3$<br>$C_8H_{11}O_2$<br>$C_9H_9O_3$<br>$C_9H_{11}O_4$	11	Detected	Detected	2a
Demethyl oleuropein	$C_{24}H_{30}O_{13}$	525.1613	525.1616	4.21	4.31	163.0374<br>389.1023	$C_9H_7O_3$<br>$C_{16}H_{21}O_{11}$	14	Detected	Detected	2b

**Compound**	**Molecular Formula**	**[M-H]⁻ m/z calc.**	**[M-H]⁻ m/z exp.**	**t_R exp. (min)**	**t_R QSRR (min)**	**Fragments m/z**	**Elemental Formula**	**Peak score A/I**	**Leaf**	**Drupe**	**Level Ident.**
Fraxamoside	$C_{25}H_{30}O_{13}$	537.1613	537.1616	4.35	5.16	133.0293<br>161.0238<br>165.0547<br>179.0335<br>205.0501<br>221.0472<br>235.0597<br>323.0777	$C_8H_5O_2$<br>$C_9H_5O_3$<br>$C_9H_9O_3$<br>$C_9H_7O_4$<br>$C_{11}H_9O_4$<br>$C_{11}H_9O_5$<br>$C_{12}H_{11}O_5$<br>$C_{15}H_{15}O_8$	9	Detected	Detected	2a
10-Hydroxy-10-Methyl oleuropein aglycone	$C_{20}H_{24}O_9$	407.1347	407.1350	6.60	6.75	111.0087<br>121.0295<br>179.0351<br>241.0871	$C_5H_3O_3$<br>$C_7H_5O_2$<br>$C_9H_7O_4$<br>$C_{15}H_{13}O_3$	10	Detected	Detected	2b
10-Hydroxy decarboxymethyl oleuropein aglycone	$C_{17}H_{20}O_7$	335.1136	335.1136	4.58	5.52	85.0296<br>121.0292<br>153.0557<br>199.0613	$C_4H_5O_2$<br>$C_7H_5O_2$<br>$C_8H_9O_3$<br>$C_9H_{11}O_5$	11	Detected	Detected	2b
10-Hydroxy oleuropein aglycone	$C_{19}H_{22}O_9$	393.1193	393.1195	4.63	5.48	137.0244<br>181.0502	$C_7H_5O_3$<br>$C_9H_9O_4$	12	Detected	Detected	2b
Ligstroside	$C_{25}H_{32}O_{12}$	523.1821	523.1820	6.74	6.80	69.0340<br>89.0242<br>101.0240<br>127.0400<br>139.0032<br>165.0571<br>171.0293	C_4H_5O<br>$C_3H_5O_3$<br>$C_4H_5O_3$<br>$C_6H_7O_3$<br>$C_6H_3O_4$<br>$C_9H_9O_3$<br>$C_7H_7O_5$	11	Detected	Detected	2a

Compound	Molecular Formula	[M-H]- m/z calc.	[M-H]- m/z exp.	t_R exp. (min)	t_R QSRR (min)	Fragments m/z	Elemental Formula	Peak score A/I	Leaf	Drupe	Level Ident.
						223.0629<br>259.0966<br>291.0881<br>361.1305	$C_{11}H_{11}O_5$<br>$C_{15}H_{15}O_4$<br>$C_{15}H_{15}O_6$<br>$C_{19}H_{21}O_7$				
Ligstroside aglycone	$C_{19}H_{22}O_7$	361.1291	361.1293	6.79	6.83	69.0346<br>111.0077<br>137.0608<br>195.0663<br>259.0975<br>291.0875	C_4H_5O<br>$C_5H_2O_3$<br>$C_8H_9O_2$<br>$C_{10}H_{11}O_4$<br>$C_{15}H_{15}O_4$<br>$C_{15}H_{15}O_6$	21	Detected	Detected	2a
Nuzhenide	$C_{31}H_{42}O_{17}$	685.2349	685.2346	6.20	6.19	89.0241<br>135.0453<br>223.0612<br>291.0871<br>369.0825<br>453.1401<br>563.1616	$C_3H_5O_3$<br>$C_8H_7O_2$<br>$C_{11}H_{11}O_5$<br>$C_{15}H_{15}O_6$<br>$C_{16}H_{17}O_{10}$<br>$C_{21}H_{25}O_{11}$<br>$C_{23}H_{31}O_{16}$	12	-	Detected	2a
Oleoside	$C_{16}H_{22}O_{11}$	389.1089	389.1090	6.79	*	139.0032<br>165.0552<br>183.0664	$C_6H_3O_4$<br>$C_9H_9O_3$<br>$C_9H_{11}O_4$	4	Detected	Detected	2a
Oleuropein aglycone	$C_{19}H_{22}O_8$	377.1241	377.1243	7.39	6.88	95.0496<br>111.0087<br>139.0389<br>171.0286<br>191.0346<br>275.0556	C_6H_7O<br>$C_5H_3O_3$<br>$C_7H_7O_3$<br>$C_7H_7O_5$<br>$C_{10}H_7O_4$<br>$C_{14}H_{11}O_6$	19	Detected	Detected	2a

**Compound**	**Molecular Formula**	**[M-H]⁻ m/z calc.**	**[M-H]⁻ m/z exp.**	**t_R exp. (min)**	**t_R QSRR (min)**	**Fragments m/z**	**Elemental Formula**	**Peak score A/I**	**Leaf**	**Drupe**	**Level Ident.**
Secologanoside	$C_{16}H_{22}O_{11}$	389.1089	389.1088	1.28	2.79	95.0499<br>101.0244<br>139.0752<br>165.0550<br>183.0645<br>345.1100	C_6H_7O<br>$C_4H_5O_3$<br>$C_8H_{11}O_2$<br>$C_9H_9O_3$<br>$C_9H_9O_4$<br>$C_{15}H_{21}O_9$	4	Detected	Detected	2a
						**Triterpenic acids**					
Maslinic acid	$C_{30}H_{48}O_4$	471.3484	471.3481	12.86	12.58	393.3163<br>405.3158<br>423.3256<br>437.3061	$C_{28}H_{41}O$<br>$C_{29}H_{41}O$<br>$C_{29}H_{43}O_2$<br>$C_{29}H_{41}O_3$	15	Detected	Detected	2a
Oleanolic acid	$C_{30}H_{48}O_3$	455.3535	455.3536	13.91	12.69**	120.0940<br>152.1202<br>407.3313<br>441.3430	C_9H_{12}<br>$C_{10}H_{16}O$<br>$C_{29}H_{43}O$<br>$C_{29}H_{45}O_3$	16	Detected	Detected	2a
						**Coumarins**					
Aesculin	$C_{15}H_{15}O_9$	338.0643	338.0645	5.41	4.01	112.0164<br>153.0189<br>161.0248<br>171.0287<br>220.0581<br>268.0577<br>311.0761	$C_5H_4O_3$<br>$C_7H_5O_4$<br>$C_9H_5O_3$<br>$C_7H_7O_5$<br>$C_8H_{12}O_7$<br>$C_{12}H_{12}O_7$<br>$C_{14}H_{15}O_8$	16	Detected	-	2a
						**Other compounds**					

**Compound**	**Molecular Formula**	**[M-H]⁻ m/z calc.**	**[M-H]⁻ m/z exp.**	**t_R exp. (min)**	**t_R QSRR (min)**	**Fragments m/z**	**Elemental Formula**	**Peak score A/I**	**Leaf**	**Drupe**	**Level Ident.**
Elenolic acid	$C_{11}H_{14}O_6$	241.0714	241.0716	4.09	4.26	95.0494<br>121.0289<br>127.0396<br>139.0029<br>139.0410<br>149.0236<br>165.0572	C_6H_7O<br>$C_7H_5O_2$<br>$C_6H_7O_3$<br>$C_6H_3O_4$<br>$C_7H_7O_3$<br>$C_8H_5O_3$<br>$C_9H_9O_3$	24	Detected	Detected	2a
Elenolic acid 2-O-glucoside (Oleoside 11-methyl ester)	$C_{17}H_{24}O_{11}$	403.1245	403.1242	2.96	2.12	113.0248<br>119.0352<br>181.0512<br>310.1163<br>371.0900	$C_5H_5O_3$<br>$C_4H_8O_4$<br>$C_9H_9O_4$<br>$C_{15}H_{18}O_7$<br>$C_{16}H_{19}O_{10}$	11	Detected	Detected	2a
Hydroxylated form of elenolic acid	$C_{11}H_{14}O_7$	257.0667	257.0671	2.92	*	137.0603<br>181.0535	$C_8H_9O_2$<br>$C_9H_9O_4$	4	Detected	-	3
Licodione	$C_{15}H_{12}O_5$	271.0611	271.0616	7.19	7.63	83.0154<br>119.0506<br>151.0037<br>177.0160<br>187.0364	$C_4H_3O_2$<br>C_8H_7O<br>$C_7H_3O_4$<br>$C_9H_5O_4$<br>$C_{11}H_7O_3$	5	Detected	-	2a

Compound	Molecular Formula	[M-H]- m/z calc.	[M-H]- m/z exp.	t_R exp. (min)	t_R QSRR (min)	Fragments m/z	Elemental Formula	Peak score A/I	Leaf	Drupe	Level Ident.
Suspensaside	$C_{29}H_{36}O_{16}$	639.1930	639.1934	4.46	6.01	113.0244<br>127.0399<br>135.0442<br>161.0233<br>179.0335<br>241.0694<br>265.0743<br>325.0944<br>459.1525<br>479.1560<br>621.1832	$C_5H_5O_3$<br>$C_6H_7O_3$<br>$C_8H_7O_2$<br>$C_9H_5O_3$<br>$C_9H_7O_4$<br>$C_{11}H_{13}O_6$<br>$C_{13}H_{13}O_6$<br>$C_{15}H_{17}O_8$<br>$C_{20}H_{27}O_{12}$<br>$C_{23}H_{27}O_{11}$<br>$C_{29}H_{33}O_{15}$	7	-	Detected	2a

Peak Score (A/I): ratio of peak area to intensity

Level Ident.: Level of identification (Level 1 corresponds to confirmed structures with a reference standard; level 2a: evidence by spectra matching from literature or library and level 2b: diagnostic evidence where no other structure fits the experimental MS/MS information; level 3 for tentative candidate).

t_R QSRR: Predicted retention time with the in-house QSRR model [1,2]

*t_R: The retention time prediction results are not reliable and other verification methods such as MS/MS fragmentation pattern should be applied.

**t_R: The retention time prediction result is not reliable because the compound is found to be outside of applicability domain of the model [3,4].

molecules MDPI

Table S6. Percentages of increase or decrease of the analytical signals during the experimental time periods 3 to 6 min and 6 to 10 min

Compounds	% increase in analytical signal (3-6 min infusion)	% increase in analytical signal (6-10 min infusion)
**Target compounds**		
Gallic acid	99	98
Tyrosol	129	231
Vanillin	119	113
Luteolin	85	72
**Suspect compounds**		
4-Hydroxybenzoic acid	99	101
4-Hydroxyphenylacetic acid	128	119
Vanillic acid	104	107
Verbascoside	129	125
Calceolarioside	76	80
Hydroxytyrosol glucoside	125	118
Tyrosol glucoside (Salidroside)	118	117
Apigenin-7-O-glucoside	125	379
Diosmin	64	71
Luteolin-7-O-glucoside	94	103
Luteolin-7,4′-O-diglucoside	128	136
Quercetin-3-O-rutinoside (Rutin)	120	124
Vicenin-2	120	112
Demethyl oleuropein	229	127
Fraxamoside	110	104
10-Hydroxy-10-Methyl oleuropein aglycone	105	103
10-Hydroxy decarboxymethyl oleuropein aglycone	91	249
Ligstroside	128	115
Ligstroside aglycone	127	111
Oleoside	114	115
Oleuropein aglycone	122	123
Secologanoside	114	122
Elenolic acid 2-O-glucoside (Oleoside 11-methyl ester)	122	117

References

1. Aalizadeh, R.; Thomaidis, N.S.; Bletsou, A.A.; Gago-Ferrero, P. Quantitative Structure-Retention Relationship Models to Support Nontarget High-Resolution Mass Spectrometric Screening of Emerging Contaminants in Environmental Samples. *J. Chem. Inf. Model.* **2016**, *56*, 1384–1398, doi:10.1021/acs.jcim.5b00752.
2. Gago-Ferrero, P.; Schymanski, E.L.; Bletsou, A.A.; Aalizadeh, R.; Hollender, J.; Thomaidis, N.S. Extended Suspect and Non-Target Strategies to Characterize Emerging Polar Organic Contaminants in Raw Wastewater with LC-HRMS/MS. *Environ. Sci. Technol.* **2015**, *49*, 12333–12341, doi:10.1021/acs.est.5b03454.
3. Kalogiouri, N.P.; Alygizakis, N.A.; Aalizadeh, R.; Thomaidis, N.S. Olive oil authenticity studies by target and nontarget LC–QTOF-MS combined with advanced chemometric techniques. *Anal. Bioanal. Chem.* **2016**, *408*, 7955–7970, doi:10.1007/s00216-016-9891-3.
4. Kalogiouri, N.P.; Aalizadeh, R.; Thomaidis, N.S. Application of an advanced and wide scope non-target screening workflow with LC-ESI-QTOF-MS and chemometrics for the classification of the Greek olive oil varieties. *Food Chem.* **2018**, *256*, 53–61, doi:10.1016/j.foodchem.2018.02.101.
